# Cancer-Associated Fibroblasts and Tumor-Associated Macrophages in Cancer and Cancer Immunotherapy

**DOI:** 10.3389/fonc.2021.668731

**Published:** 2021-05-20

**Authors:** Hans Raskov, Adile Orhan, Shruti Gaggar, Ismail Gögenur

**Affiliations:** ^1^ Center for Surgical Science, Zealand University Hospital, Køge, Denmark; ^2^ Department of Biomedical Sciences, University of Copenhagen, Copenhagen, Denmark; ^3^ Department of Clinical Medicine, University of Copenhagen, Copenhagen, Denmark

**Keywords:** cancer-associated fibroblasts, tumor-associated macrophages, tumor microenvironment, cancer immunotherapy, cancer biology

## Abstract

Our understanding of the tumor microenvironment (TME), including the interplay between tumor cells, stromal cells, immune cells, and extracellular matrix components, is mandatory for the innovation of new therapeutic approaches in cancer. The cell-cell communication within the TME plays a pivotal role in the evolution and progression of cancer. Cancer-associated fibroblasts (CAF) and tumor-associated macrophages (TAM) are major cell populations in the stroma of all solid tumors and often exert protumorigenic functions; however, the origin and precise functions of CAF and TAM are still incompletely understood. CAF and TAM hold significant potential as therapeutic targets to improve outcomes in oncology when combined with existing therapies. The regulation of CAF/TAM communication and/or their differentiation could be of high impact for improving the future targeted treatment strategies. Nevertheless, there is much scope for research and innovation in this field with regards to the development of novel drugs. In this review, we elaborate on the current knowledge on CAF and TAM in cancer and cancer immunotherapy. Additionally, by focusing on their heterogenous functions in different stages and types of cancer, we explore their role as potential therapeutic targets and highlight certain aspects of their functions that need further research.

## Introduction

Originating from the neighboring healthy tissues and recruited from the circulation, a multitude of proliferating non-neoplastic cells such as fibroblasts, macrophages, immune cells, and endothelial cells contribute to carcinogenesis within the tumor microenvironment (TME) (1). Cancer-associated fibroblasts (CAF) and tumor-associated macrophages (TAM) are the major cell populations within the stroma of all solid tumors in which they often exert protumorigenic functions. Although their precise interactions remain to be elucidated, CAF and TAM strongly modulate disease progression, therapy resistance, and clinical outcomes (2–7) and may function in synergy.

Targeting the cytokines, inhibitory immune checkpoint ligands expressed by CAF and TAM, and antiphagocytic signaling by tumor cells have shown some efficacy in preclinical trials. The results of clinical trials are nonetheless ambiguous. Antibodies, chemokines, and chemokine ligands that interfere with CAF/TAM interactions, and their combinations hereof, are highly prioritized in experimental clinical regimens that are aimed at modulating the TME (8).

## The Fibroblast

Fibroblasts can be clearly identified and characterized by their elongated morphology, the lack of epithelial, endothelial, leukocytic, and malignant-cell markers, and the positivity for mesenchymal markers such as vimentin. Under normal circumstances, fibroblasts are present in abundance in the connective tissues in a dormant state, transiently being activated during periods of tissue remodeling and repair. They are involved in the production of extracellular matrix (ECM) and modulation of inflammation, as well as the proliferation and differentiation of epithelial cells.

Well-established fibroblast-activating signals include inflammatory mediators, transforming growth factor-beta (TGF-β), and lysophosphatidic acid which increase the activity of SMAD transcription factors and drive the expression of alpha-smooth muscle actin (α-SMA) that provides the fibroblast with a highly contractile phenotype (usually known as myofibroblast or α-SMA^+^ fibroblast). The activated fibroblasts produce chemokines and cytokines to regulate the communication with other mesenchymal, epithelial, and immune cells (9). Importantly, all of these functions are utilized and enhanced in cancer (10, 11).

## Cancer-Associated Fibroblasts

### Structure and Functions

Within a tumor, the mesenchymal cells that comply with the aforementioned definitions above, are generally referred to as CAF. Compared with regular fibroblasts, they tend to be slightly larger with darker nuclei and branched cytoplasm. CAF may differentiate from quiescent fibroblasts and bone marrow-derived mesenchymal stem cells or trans-differentiate from epithelial cells, smooth muscle cells, pericytes, and adipocytes (12).

CAF are present during all stages of solid malignancies (13) and their functional impact on the biology of cancer is assumed to be similar across all tumor types (14).

CAF are the predominant cell type in the tumor stroma and they contribute to the proliferative, pro-inflammatory, immunosuppressive, angiogenic, pro-invasive, and pro-metastatic TME that is required for the evolution and progression of cancer (15).

Inflammatory mediators such as TGF-β, interleukin (IL)-1, and IL-6 produced by tumor cells and non-malignant stromal cells promote CAF activation and contribute to a pro-inflammatory profile, that directly support carcinogenesis (16). The activation of specific transcriptional programs and the lack of negative feedback mechanisms launch CAF into self-sustaining trajectories (17, 18).

Additionally, CAF drive the epithelial-mesenchymal transition (EMT), whereby cancer cells lose polarity and adhesion molecules and gain the motility necessary for dissemination (19). Despite the overall pro-tumorigenic effects, functional dualities have been observed. A hypothesis is that initially CAF are tumor suppressive but as cancer evolves they transform into pro-tumorigenic cells (20).

### Heterogeneity of CAF Subtypes

Within a multi-clonal solid tumor, CAF are differentially exposed to a multitude of tumor secreted factors (TSF) explaining their heterogeneity. However, the essential molecular mechanisms underlying the activation and pro-tumorigenic activities of fibroblasts may be common to various cancers, which present a manifold of targets for innovative CAF-targeted therapies. Signaling cascades mainly involve the Wnt/β-catenin, TGF-β, epidermal growth factor receptor, JAK/STAT, and Hippo pathways.

Several studies have characterized distinct CAF subgroups that differentially express the CAF markers, e.g. α-SMA, fibroblast activation protein (FAP), and platelet-derived growth factor receptor (PDGFR), and show that CAF subpopulations may have various and even opposing functions. Tumor-suppressive CAF populations have been characterized by activated Hedgehog signaling pathways in mouse models of colon, pancreatic, and bladder cancers. However, the full complement of CAF populations remains unclear, and more detailed classifications and functions of CAF subtypes are needed (21–25).

In a mouse model of pancreatic ductal adenocarcinoma (PDAC), the ablation of CAF led to enhanced hypoxia, EMT, increased vascularity, cancer cell proliferation, and disease progression demonstrating that CAF to some extent can restrain tumor growth (26, 27). Similarly, an initial expansion of local fibroblasts circumscribing early or premalignant lesions in response to tissue neoplasia was observed in mouse models and human tissue studies (14, 28, 29).

Thus, the TME comprises a heterogeneous population of CAF subtypes or clusters with different functions associated with immunomodulation, immunosuppression, and immunotherapy resistance (30).

Furthermore, in a mouse model on early and late PDAC stages, fibrosis associated with type I collagen provided a protective response from the host rather than a pro-tumorigenic response (26).

These results demonstrate that at least some stromal constituents may restrain rather than promote tumor progression and illustrate the high degree of temporal differentiation plasticity within the diverse cell populations of tumors. This may also explain the conflicting reports regarding antitumor and pro-tumor functions of CAF.

In a preclinical trial on lung cancer, the depletion of CAF significantly reduced the number of metastases (31, 32). To establish the clinical relevance of primary tumor CAF in the formation of metastasis, this research group examined human brain metastases (since the normal brain is devoid of fibroblasts) from lung, breast, kidney, and endometrium, and found a distribution of activated CAF within these metastases. These findings support the view that the CAF shed from the primary tumor, together with cancer and non-tumor cells from the TME, survive during the blood circulation and proliferate at the metastatic site (31).

With respect to human PDAC specimens, the patients with a higher expression of FAP were found to be associated with shorter disease-free survival and overall survival when compared to those with low FAP expression (33). The immune suppression caused by FAP^+^ CAF is mediated by the CXCL12 receptor CXCR4 that excludes T cells from the tumor. Notably, CXCR4 inhibition leads to an elimination of tumor cells by a rapid accumulation of cytotoxic CD8^+^ T cells (34). Moreover, the deletion of FAP^+^ CAF using a FAP-targeted immune-based therapeutic approach or a genetic ablation approach inhibited cancer growth in murine PDAC models (32, 35). Thus, the inhibition of CAF-induced pro-tumorigenic signals is a highly attractive future strategy to improve outcomes in pancreatic cancer.

In human triple-negative breast cancer, a subset of CAF with myofibroblast characteristics (myCAF) (α-SMA^+^/FAP^+^ or S1 CAF) was identified as a key player in immunosuppression through the attraction of T_regs_ and inhibition of effector T cell proliferation (36) and it was hypothesized that targeting the CAF-S1-mediated immunosuppression could enhance anti-tumor immunity.

In PDAC, CAF are linked to worse overall survival. PDAC is infamous for the abundance of fibrotic ECM with the majority of the tumor volume being composed of α-SMA^+^ CAF. Preclinical and clinical trials targeting stromal α-SMA^+^ CAF, however, resulted in an apparent, paradoxical acceleration in disease progression and reduction in survival, halting clinical trials and adding further layers of complexity to CAF functions (26, 37).

Another study on murine models of lung carcinoma and PDAC revealed that the deletion of FAP led to a significant reduction in CAF infiltration and tumor tissue necrosis, and an increase in infiltration of CD8^+^ T cells (38). Moreover, in murine models of breast and colon cancer, the administration of a DNA-based vaccine targeting FAP induced the killing of CAF by CD8^+^ T cells and lead to a substantial increase in the uptake of chemotherapeutic agents by otherwise multi-drug-resistant cancer cells (39). Further, in immunocompetent mice, the cell transfer of FAP-specific chimeric antigen receptor T cells boosted host immunity and arrested pancreatic tumor growth; however, it also led to significant lethal toxicity and cachexia (40). These examples indicate that specific CAF subsets could be potential targets for improving immunotherapy. Future studies are needed to develop targeted therapies aimed at specific CAF populations (41).

### Secreted Factors and Exosomes in CAF-Tumor Cells Interplay

**Figure 1:**
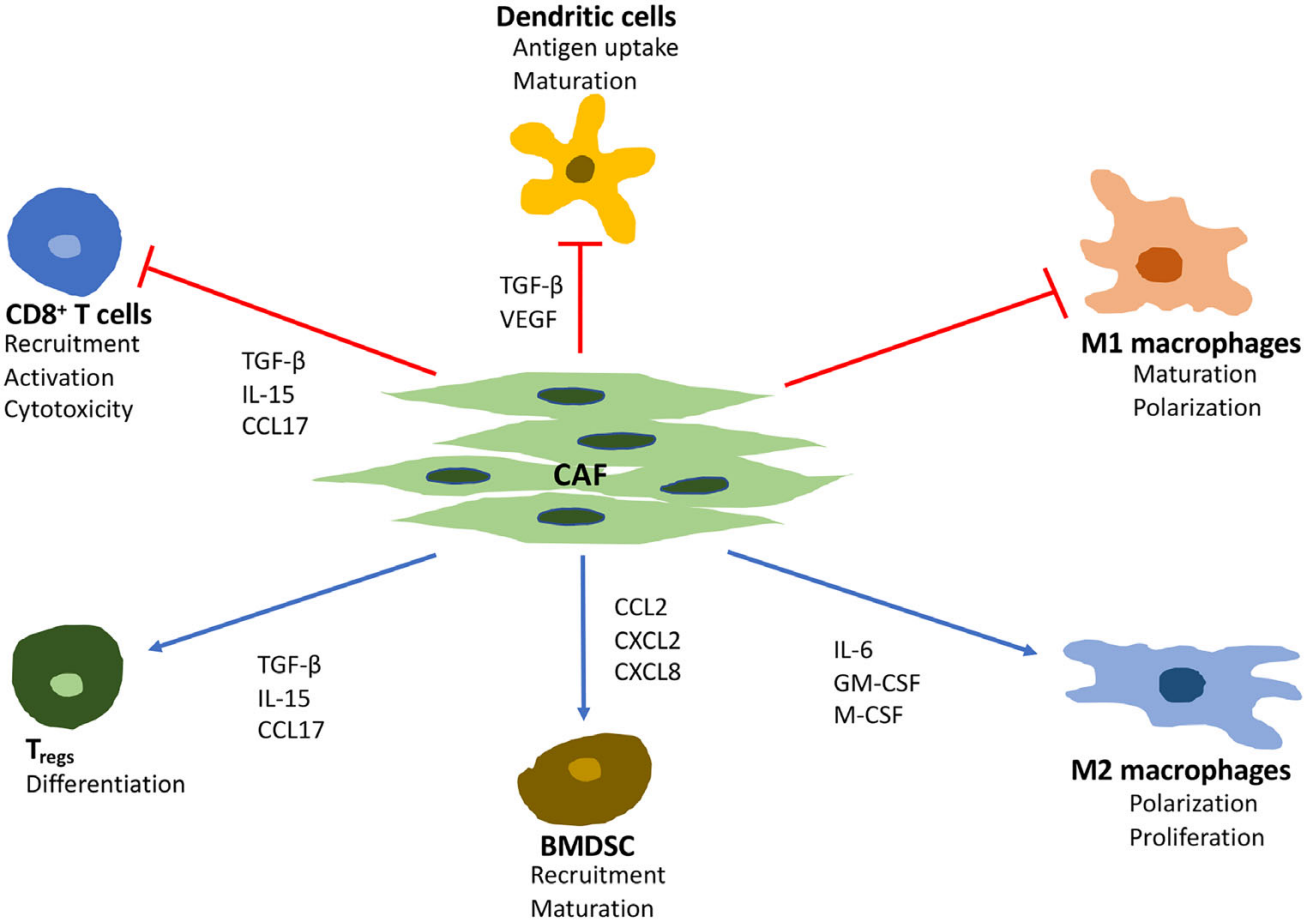
Major effects of CAF on immune cells in the tumor microenvironment. TGF-β, transforming growth factor beta; VEGF, vascular endothelial growth factor; IL, interleukin 6; GM-CSF, granulocyte-macrophage colony-stimulating factor; M-CSF, macrophage colony stimulating factor; CCL, C-C motif chemokine ligand; CXCL, C-X-C motif ligand.

The cytokines and chemokines produced by CAF may have both immunosuppressive and immuno-activating effects on various leukocytes, including CD8^+^ T cells, immunosuppressive regulatory T cells (T_regs_), and macrophages (**Figure 1**). However, the consensus is that the overall effects of CAF are immunosuppressive (14). IL-6, CXC-chemokine ligand (CXCL) 9, and TGF-β, which are produced by CAF, have well-established roles in suppressing anti-tumor T cell responses (34). This is also supported by an inverse association between CAF and CD8^+^ T cell cytotoxicity.

The staining of the inhibitory immune-receptor ligand programmed death-ligand 2 (PD-L2) and tumor necrosis factor-alpha (TNF-α) ligand OX40L in human breast cancer sections revealed T lymphocytes at the surface of CAF. This confirmed that subsets of CAF attract and retain T lymphocytes at the periphery of the tumor through distinct mechanisms involving chemokine signaling (chemokine ligand [CCL]-11, CXCL12–14), cell adhesion molecules, activation of inhibitory immune checkpoints, and CD8^+^ T cell anergy (36).

In a murine PDAC model, it was demonstrated that CAF, programmed by TGF-β to express a leucine-rich protein (LRRC15), were associated with a poor response to anti–PD‐L1 therapy (42). Additionally, CAF are a source of various growth factors including TGF-β, vascular endothelial growth factor (VEGF), fibroblast growth factor 5, growth differentiation factor 15, hepatocyte growth factor and insulin-like growth factor (43, 44). The secretion of pro-stemness paracrine factors such as insulin-like growth factors, inflammatory cytokines (IL-6 and IL-8), and chemokines (CCL2 and CCL5) promotes the conversion of cancer cells into cancer stem cells and reinforce the stemness of existing cancer stem cells (45–47). Moreover, the secretion of IL-6 make CAF an important mediator of EMT in cancer cells (48, 49).

Exosomes are extracellular vesicles released by all cell types and are found in all bodily fluids (50). They contain genetic material, proteins, and lipids and are essential for intercellular communication. The activation, recruitment, and conversion of fibroblasts into activated CAF depend on TSF and tumor-secreted exosomes (TSE) containing various oncogenic molecules such as microRNAs (miRs), fusion gene mRNAs, long non-coding RNAs, mutated DNA fragments, and a manifold of cell‐signaling molecules (51). The circulating levels of exosomal miRNA accurately reflect disease progression and could serve as a prognostic tool among various cancers following resection of the primary tumor (52–58).

In addition to TSF, TSE and CAF-derived exosomes (CAFEx) secreted by tumor cells and CAF, respectively, in the primary tumor are critical mediators of cancer cell-immune cell communication and they drive the formation of pre-metastatic niches (PMN) (59). Moreover, CAF may enter the circulation and promote the development of PMN and subsequent metastatic lesions (60, 61).

Integrins (ITG) are known to determine tumor cell organotropism. In a mouse model, CAF promoted lung metastasis by the construction of PMN *via* CAFEx. CAFEx-derived ITGα_2_β_1_ were found to home to the lung fibroblasts and subsequently activate the TGF-β signaling pathway. To prepare for subsequent colonization of the lung tissue by extravasating circulating tumor cells (CTC), the lung microenvironment is remodeled by the activated lung fibroblasts (58). Surface ITG guide the TSE to organ-specific ECM ligands (collagen, fibronectin, fibrinogen, and E-cadherin) in the target organs, e.g. ITGα_6_β_1_ and ITGα_6_β_4_ adhere to the epithelial cells and fibroblasts in the lung and ITGα_v_β_5_ binds to resident liver macrophages (Kupffer cells) and upregulate the genes for cell migration and S100 protein (62). Organ-specific TSE have been identified for 28 different metastatic cell lines. Furthermore, TSE comprising TGF-β and PDGF mediate the activation, differentiation, and recruitment of CAF through all stages of all solid cancers (13).

In early-stage colorectal cancer (CRC), TSE were found to promote highly proliferative and angiogenic CAF, while those from late-stage metastatic CRC cell lines were observed to induce highly invasive CAF which, through the secretion of ECM-degrading proteases and increased expression of the pro‐invasive modulators of membrane protrusion, enabled the penetration of ECM (51).

In addition, TSE alter CAF metabolism and induce the production of CAFEx containing nutrient metabolites (amino acids and tricarboxylic acid cycle intermediates) that fuel the tumor cells and increase their survival (31, 63). A study on breast-cancer cell lines revealed that TSE containing miR-105 could re-program CAF metabolism and enable them to increase glucose metabolism when nutrient levels were sufficient as well as detoxify metabolic wastes into energy-rich metabolites when nutrients were scarce (64).

As shown in PDAC, lactate produced by cancer cells promotes extensive epigenomic reprogramming of CAF (65). In CRC, and during protein deprivation, CAF accumulate fatty acids, phospholipids, and fatty acid synthetase. The uptake of lipid metabolites by the CRC cells secreted by CAF seem to be essential for their migration (66).

Another potent promotor of malignancy is the heat shock factor 1 which is frequently activated in CAF. It drives a program that supports the survival and metastatic potential of cancer cells by inhibiting apoptosis and promoting migration. The activation of heat shock factor 1 has been associated with poor outcomes in CRC, lung-, breast-, and hepatocellular carcinoma (HCC) (67).

Of the important players, the gene that deserves mentioning is the HMG-box 2 (SOX2). It codes for transcription factors controlling the expression of several genes involved in early embryonic development. The upregulated stromal SOX2 drives the reprogramming of colonic fibroblasts that results in enhanced β-Catenin and TGF-β signaling in CRC cells supporting cancer progression. Nonetheless, the precise mechanism remains to be determined (68).

The subset of CAF with myofibroblasts characteristics (myCAF) mediate a chronically deranged wound healing program in tumors and play a key role in the development of a continuously evolving fibrotic stroma. myCAF are highly responsive to chemokines and metabolically and morphologically distinctive from CAF. When activated, their proliferation rate drops and the production of ECM components increases dramatically. The cytoplasmic microfilaments of myCAF connect to the extracellular fibronectin domains, creating very contractile mechanisms. The following extracellular deposition of collagen reinforces and stiffens the ECM (69).

Not only does it contribute to the increasing stromal density, but the remodeling of the stroma by CAF-produced matrix-enzymes also provides tracks for cancer cell invasion and migration (14). The stromal stiffness results in increased interstitial pressure, abnormal vasculature, collapsed blood vessels, hypoxia, and acidity which lead to inefficient drug delivery and reduced response to therapy. These physical and chemical barriers are hostile to cytotoxic immune cells such as CD8^+^ T cells and natural killer (NK) cells (70).

### CAF and Circulating Tumor Cells (CTC)

The presence of CAF in the circulation of cancer patients and their levels in the peripheral blood correlates with cancer progression and worse prognosis. Notably, the high levels of CAF-CTC aggregates in the blood samples from patients should be considered an important marker of worse clinical outcomes (71). For instance, CTC have higher viability in the blood stream when accompanied by stroma cells that also provide an advantage with respect to early survival and growth of tumor cells at the metastatic site (31). Traveling in clusters with macrophages, immune cells, and platelets, CAF support, shield, and increase the survival of CTC. Adjoining neutrophils may aid in the survival of CTC through the suppression of leukocyte activation (72). Through strong intercellular adhesions, CAF maintained the viability and proliferative capacity of CTC in cellular aggregates in presence of high levels of hemodynamic forces (> 1,000 dyn/cm^2^). This protective role was observed in prostate cancer, usually spreading through blood vessels rather than the lymphatic system (61).

Only a minority of CTC travel in clusters; however, in a mouse model, it was estimated that the probability of metastasis formation originating from clusters (and especially those of oligoclonal tumor cell groupings) is fifty times higher compared with that originating from a single CTC (73).

As EMT of tumor cells may proceed within the clusters, the association between neutrophils and CTC drives tumor cell mitosis and expands the metastatic potential of CTC (74). Upon arrival in the PMN, tissue-resident fibroblasts contribute to the mesenchymal-epithelial transition (MET). Thus, CAF are considered key players in promoting the survival of CTC.

### Targeting CAF-Associated Pathways

To revert CAF to a quiescent state by targeting the activation pathways is an appealing concept. CAF-secreted Wnt2 accelerates the Wnt/β-catenin signaling pathway which corresponds with the absence of CD8^+^ T cells. The effects of vitamin D seen in epidemiological studies of PDAC and CRC are partly related to the reduced CAF-related Wnt/β-catenin signaling which was relayed by vitamin D metabolites (**Table 1**) (75).

**Table 1:** Clinical trials targeting Wnt/β-catenin signaling related to CAF in different types of cancer.

Cancer type	Trial number	Target	Mechanism of action	Treatment/Intervention
CRC	NCT04094688	CAF-related Wnt/β-catenin signaling	Wnt pathway: Vitamin D3 promotes the upregulation of DKK-1 (tumor suppressor) and downregulation of DKK 4 β catenin: Vitamin D3 promotes VDR-dependent inhibition of β-catenin (1)	High dose vitamin D3 + FOLFOX/FOLFIRI + Bevacizumab
PDAC	NCT03520790			Gemcitabine + Nab-paclitaxel + Paricalcitol IV/oral
Melanoma	NCT01748448			Vitamin D
Urothelial cancer	NCT04197089			Vitamin D
Prostate cancer	NCT03103152			High/Low dose Aspirin + Vitamin D
Gynecologic cancers	NCT03192059			Vitamin D + Aspirin + Cyclophosphamide + Lansoprazole + Pembrolizumab + Radiation + Curcumin
Breast cancer	NCT02786875			Low glycemic diet, Physical activity, and Vitamin D

FOLFOX: leucovorin, 5-fluorouracil, and oxaliplatin; FOLFIRI: leucovorin, 5-fluorouracil, and irinotecan

CRC, colorectal cancer; PDAC, pancreatic ductal adenocarcinoma; CAF, cancer-associated fibroblasts; VDR, vitamin D receptor; DKK 1, DICKKOPF 1.

(1) Pendás-Franco, Natalia et al. “Vitamin D and Wnt/beta-catenin pathway in colon cancer: role and regulation of DICKKOPF genes.” Anticancer research vol. 28,5A (2008): 2613-23.

Alternatively, targeting CAF-derived cytokines and chemokines (e.g. CXCL, IL-6, and TGF-β) could improve anticancer efficiency in combination with immunotherapy. Several IL-6 inhibitors such as sarilumab and tocilizumab that are already approved for autoimmune and myeloproliferative disorders, are being investigated for their role in anticancer therapy either alone or in combination.

Anti-TGF-β in combination with anti-PD-L1 antibodies inhibited TGF-β signaling in CAF and facilitated T cell penetration into solid tumors (76). A summary of RCT examining the effects of targeting IL-6 and TGF-β have been presented in **Table 2**. The complexity and incomplete understanding of CAF functions necessitate further research before anti-CAF targeted therapy can be integrated into clinical practice.

**Table 2:** Clinical trials targeting CAF associated pathways involving IL-6 and TGF-β in different cancers.

Cancer type	Trial number	Target	Mechanism of action	Treatment/Intervention
Pancreatic cancer	NCT02767557	IL-6	Anti-IL-6 antibody	Tocilizumab + Nab-paclitaxel + Gemcitabine
Melanoma	NCT03999749	IL-6		Tocilizumab + Nivolumab + Ipilimumab
Prostate cancer	NCT03821246	IL-6		Tocilizumab + Atezolizumab + Etrumadenant
Esophageal cancer	NCT04595149	TGF-β + PD-L1	Bifunctional antibody against 3 isoforms of TGF-β and PD-L1 (1)	Paclitaxel + Carboplatin + Bintrafusp alfa + Radiotherapy
Head and neck cancer	NCT04247282	TGF-β + PD-L1		Bintrafusp alfa alone/+ TriAd vaccine + N-803
HPV-associated cancers	NCT04432597	TGF-β + PD-L1		PRGN-2009 alone/+ Bintrafusp alfa

Bintrafusp alfa, Anti-PD-L1/TGF-Beta Trap; N-803, IL-15 super agonist; TriAd vaccine, novel agent targeting 3 human tumor-associated antigens-CEA, MUC1, and brachyury; PRGN-2009, HPV vaccine.

IL, interleukin; TGF-β, transforming growth factor β; PD-L1, programmed death-ligand 1; HPV, human papillomavirus.

(1) Lind, Hanne et al. “Dual targeting of TGF-β and PD-L1 via a bifunctional anti-PD-L1/TGF-βRII agent: status of preclinical and clinical advances.” Journal for immunotherapy of cancer vol. 8,1 (2020): e000433. doi: 10.1136/jitc-2019-000433.

## The Macrophage and Its M1 and M2 Subtypes

Representing another major stromal cell population, macrophages are remarkable, heterogenic, and versatile cells. These cells are capable of switching functions and phenotypes, depending on their unique microenvironment (77). They engulf tissue and microbial debris; orchestrate inflammatory processes (78); and contribute to tissue remodeling, angiogenesis, and homeostasis. The conventional binary model distinguishes between the M1 and M2 macrophages.

The M1 subtype consists of classically activated, pro‐inflammatory macrophages with bactericidal, tumor‐suppressive, and anti-angiogenic functions. They express inducible nitric oxide synthase (CD86 and CD169) and are activated through their pattern recognition receptors upon recognition of damage- or pathogen-associated molecular patterns such as bacterial lipopolysaccharides and DNA damage. They produce inflammatory cytokines (e.g. IL‐1β, IL‐6, IL‐12, IL‐23, and TNF‐α), proliferate, and self-renew in a macrophage colony-stimulating factor 1 (M-CSF1)- and granulocyte-macrophage (GM)-CSF-dependent manner (79).

The M2 subtype, the alternatively activated macrophages expressing CD163, CD206, and CD204, are commonly known as TAM. They are characterized by the production of anti‐inflammatory, immunosuppressive chemokines and cytokines, such as IL-4, IL-6, IL-8, IL-10, IL-13, and TGF-β (80, 81), and are devoid of cytotoxic activity. They produce various growth factors, such as basic fibroblast growth factor (b-FGF), placental growth factor, insulin-like growth factor, epidermal growth factor (EGF), VEGF, and PDGF (82).

It should be emphasized that macrophages are extremely plastic. Many context- and tissue- dependant phenotypes on the spectrum between M1 and M2 exist, depending on multiple factors of stimulation, and these in-between phenotypes are not captured by the classical nomenclature. A more comprehensive classification system that takes the dynamic nature of macrophages into account has been proposed but so far not adopted in the literature (83).

Although their origin is still debated, it is generally believed that macrophages originate *via* common dendritic cell precursors in blood, spleen, and from bone marrow hematopoietic stem cell-derived progenitors with myeloid restricted differentiation. Embryonic precursors may seed tissues already in the fetal period and become tissue-resident macrophages (84). Attracted by chemokines, macrophage progenitors enter the circulation from reservoirs in the bone marrow and spleen. They leave the peripheral blood flow and migrate to tissues where local growth factors and cytokines control their differentiation (85).

## Tumor-Associated Macrophages

**Figure 2:**
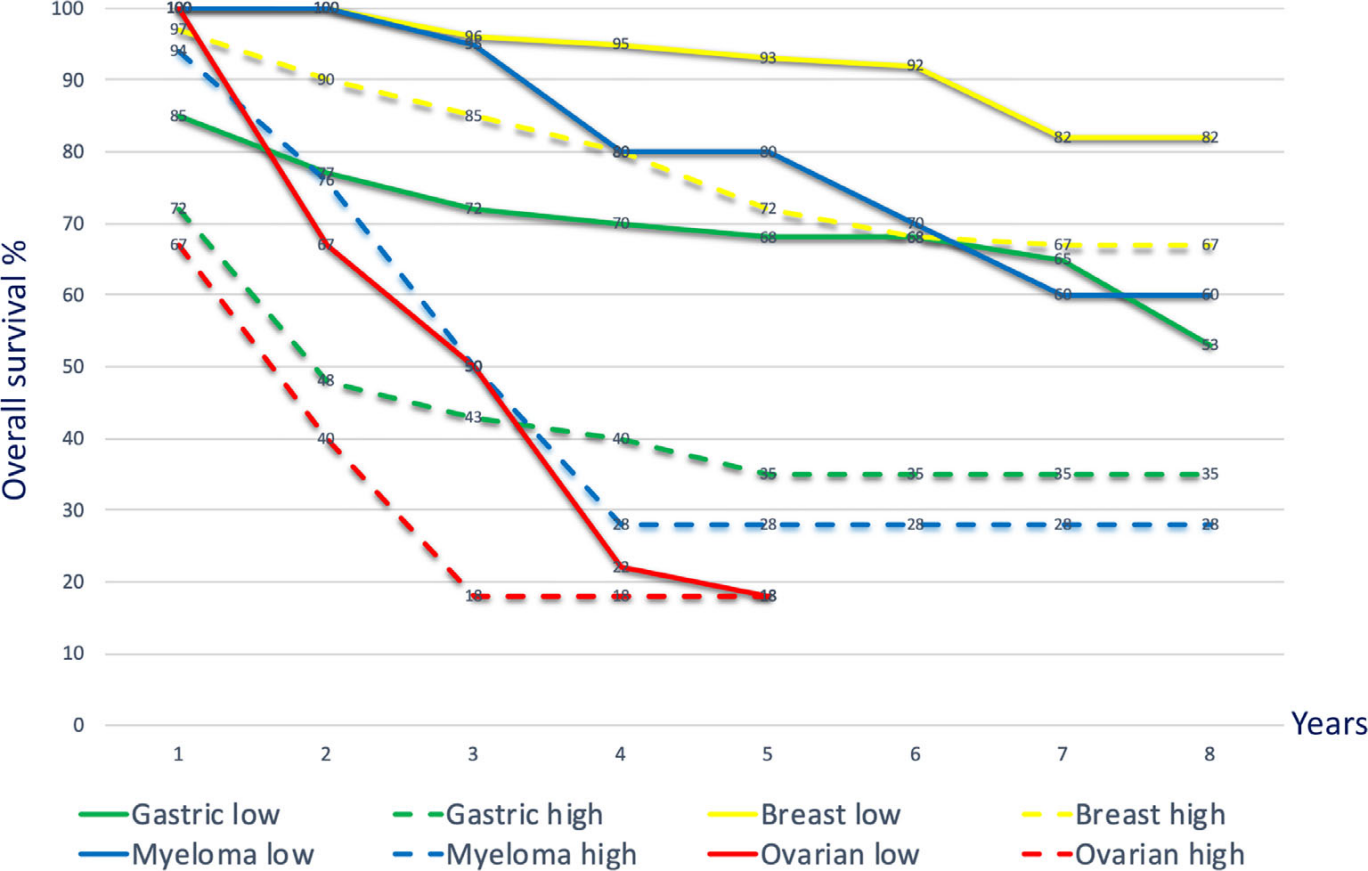
Kaplan–Meier curves depicting overall survival for high and low TAM densities across different cancer types. Overall survival curves, merged data, various cancers. Gastric cancer: Kaplan–Meier overall survival curves for gastric cancer patients with high TAM density (> 671 cells in five 400x microscopic fields; green dotted line) and low density (< 671 cells in five 400x microscopic fields; green solid line). The TAM density in the tumor tissue was negatively associated with overall survival [p=0.0073; (86)]. Breast cancer: Kaplan–Meier curves showing significant correlation (p<0.001) with overall survival according to the numbers of M2 TAM (CD163 high: yellow dotted line; CD163 low: yellow solid line) (87). Multiple myeloma: overall survival outcome based on low and high CD163 TAM (≤ 55 per high power microscopic field; dotted blue line) showing significant survival difference (p<0.001) (88). Ovarian cancer: Kaplan–Meier survival curves comparing high and low M1 (CD80)/M2 (CD163) ratios in patients with ovarian cancer. Patients with an M1/M2 ratio ≥ 1.4 (solid red line) showed a significantly higher overall survival (p=0.02) than those with an M1/M2 ratio < 1.4 (dotted red line) (89).

The level of infiltrating TAM correlates with tumor progression and reduced survival in patients (**Figure 2**). Growth factors and immunosuppressive cytokines produced by TAM can enhance motility, intravasation, and invasion of tumor cells, as well as stimulate angiogenesis and prevent attacks by T cells and NK cells (90, 91) as observed in various tumor types including carcinomas, sarcomas, and lymphomas (92–94). The recruitment of macrophages and their differentiation into TAM are primarily promoted by TSF and CAF-derived factors such as M-CSF1, GM-CSF, CCL2, VEGF, IL-6, and IL-8 (95); and are related to local anoxia, acidity, and inflammation. The infiltration into the TME is determined by CC chemokines such as the C-C motif ligands CCL2, CCL11, CCL16, and CCL21 produced by local lymphatic endothelial cells and stromal cells as demonstrated in breast-, lung-, oesophageal-, ovarian-, and cervical cancers (96, 97). Especially CCL2 exhibits strong chemotactic activity for macrophages. Producing CCL2 themselves, macrophages recruit macrophages in a feed-forward loop.

Homing towards increasing gradients of chemotactic molecules, TAM massively infiltrate hypoxic/necrotic regions of tumors and survive by shifting their metabolism towards glycolysis (98). Hypoxic TAM express the transcription factor hypoxia-inducible factor 1α and secrete VEGF, b-FGF, PDGF, cyclooxygenase-2, prostaglandin E2, and MMPs (99, 100). In response to hypoxia, TAM also overexpress PD-L1, PD-L2, and cytotoxic T-lymphocyte-associated protein 4 ligands that contribute to immune cell dysfunction and limit the effects of checkpoint inhibitors (101, 102). Furthermore, the high levels of IL‐10 and TGF‐β produced by TAM block T cell proliferation and T cell cytotoxicity, while activating T_regs_ (92, 103, 104).

### Exploring TAM in Different Cancer Types

Activated TAM are significant prognostic biomarkers for breast cancer, PDAC (105), non-small-cell lung cancer (106), gastric cancer (107), HCC (108), and stage II colon cancer (109).

In breast cancer, TAM produce metalloproteinases (MMP) and cathepsins which degrade the ECM and release angiogenic factors stored in the ECM. TAM-derived MMP-2 and MMP-9 have been correlated to a worse prognosis (110). Using human metastatic breast cancer cells, it was demonstrated that these cells stimulate TAM with M-CSF1 and in turn, TAM supply EGF to them. This paracrine feed forward mechanism between tumor cells and TAM facilitates the dissemination, intravasation, and metastatic spread of cancer (111, 112).

In gastric cancer, TAM-derived exosomes that are rich in miRNA, lncRNA, and specific proteins contribute to tumor cell dissemination. Mass spectrometric analysis revealed that these exosomes activated mitogenic signaling through the phosphatidylinositol 3-kinase (PI3K)/AKT pathway in tumor cells, inducing EMT and increasing the metastatic potential (113).

In PDAC, TAM-derived exosomes reportedly contribute to the resistance of tumor cells to gemcitabine. Using a genetic mouse model of PDAC and electron microscopy analyses, it was demonstrated that TAM exosomes are selectively internalized by tumor cells indicating that TAM and tumor cells communicate closely with each other. Furthermore, it was shown that the sensitivity of PDAC to gemcitabine was significantly reduced by the exosomal TAM-derived miR-365 (114).

In non-small-cell lung cancer tissue samples from 104 patients, M1 macrophages and TAM were identified using multiplex immunofluorescence staining. TAM predominated over M1 macrophages in number and proximity to tumor cells, which was linked with tumor cell survival, particularly in the hypoxic regions (109).

In stage II colon cancer, postoperative adjuvant chemotherapy generally has limited effect, with an improved survival rate of less than 5% at 5 years after surgery (115, 116). In a clinical study on human stage II colon cancer, a high density of CD206^+^ TAM was significantly associated with poor differentiation and worse disease-free survival. A high CD206/CD68 ratio (CD68 being an unspecific marker for the macrophage lineages) was significantly associated with poor differentiation, T4 stage, and lymphatic/vascular/perineural invasion. This ratio was a more reliable prognostic factor than CD206^+^ TAM density and other traditional clinicopathologic high-risk factors. Notably, the CD206/CD68 ratio identified patients with a low and high risk of tumor recurrence and effectively predicted which patients would benefit from adjuvant chemotherapy (117).

### Targeting TAM-Associated Pathways

The field of research exploring the mechanisms by which TAM impact the tumor progression and lower the response to anticancer therapies is very active, and it includes several pharmacological strategies to target TAM. While some strategies revolve around blocking recruitment and depleting TAM through direct inhibition using small molecules and monoclonal antibodies, others focus on reprogramming of TAM.

With respect to TAM recruitment, it has been demonstrated that the blockade of CCR2 suppresses the accumulation of TAM in tumors. CCR2 inhibitors and anti-CCL2 antibodies (CNTO 888) have demonstrated efficacy in reducing tumor growth and metastasis in several pre-clinical murine models (118, 119).

It has been reported across multiple murine tumor- and metastasis models that CCR2 antagonism in combination with anti-PD-1 therapy lead to sensitization and enhanced tumor response over anti-PD-1 monotherapy (120). Additionally, in a clinical trial on PDAC, an objective tumor response was observed in 16 of the 33 patients (49%) receiving a CCR2 inhibitor (PF-04136309) plus FOLFIRINOX, compared to FOLFIRINOX alone (**Table 3**) (121). CCR5 is another receptor which is highly upregulated in metastatic cancers, and a study in mice showed promising response upon treatment with CCR5 antagonist, maraviroc (118, 122).

**Table 3:** Clinical trials targeting CCR2-CCL2 axis and CCR2/CCR5 in TAM.

Cancer type	Trial number	Target	Mechanism	Treatment/Intervention
Metastatic PDAC	NCT02732938	CCR2	PF-04136309 binds to CCR2 and inhibits interaction between CCR2 and CCL2	PF-04136309 + Nab-paclitaxel + Gemcitabine
Locally advanced PDAC	NCT01413022			PF-04136309 + FOLFIRINOX
Solid tumors, Bone metastases	NCT01015560	CCR2	Monoclonal antibody	MLN1202
Locally advanced PDAC	NCT03767582	CCR2 + CCR5	BMS-813160 is a small-molecule dual antagonist of CCR2 and CCR5	BMS-813160 + SBRT + Nivolumab +/- GVAX
CRC and PDAC	NCT03184870			BMS-813160 alone or combined with: Nivolumab, Gemcitabine, Leucovorin, Irinotecan, Nab-paclitaxel, 5-FU
PDAC	NCT03496662			BMS-813160, Nivolumab, Gemcitabine, Nab-paclitaxel
NSCLC, HCC	NCT04123379			BMS-813160 + BMS-986253 + Nivolumab
Advanced RCC	NCT02996110			Nivolumab + Ipilumab/Relatlimab/BMS-986205/BMS813160
Solid tumors	NCT00537368	CCL2	Anti-CCL2 recombinant monoclonal antibody	CNTO 888 (discontinued)

FOLFIRINOX, 5-fluorouracil, leucovorin, irinotecan, and oxaliplatin; SBRT, stereotactic body radiotherapy; 5-FU, 5-fluorouracil; GVAX, granulocyte-macrophage colony-stimulating factor (GM-CSF) gene-transfected tumor cell vaccine; PDAC, pancreatic ductal adenocarcinoma; CRC, colorectal cancer; HCC, hepatocellular carcinoma; RCC, renal cell carcinoma; NSCLC, non-small-cell lung cancer; CCR2/5, C-C chemokine receptor type 2/5; CCL2, chemokine (C-C motif) ligand 2.

Furthermore, several trials investigating the effect of dual inhibition of CCR2 and CCR5 in patients with locally advanced pancreatic cancer, CRC, HCC, advanced renal cell carcinoma and non-small-cell lung cancer are underway (**Table 3**).

Another strategy is to deplete TAM by pharmacological blockade of CSF and its receptor CSF-1R, in mono- or combination therapy, preferentially in patients with advanced solid tumors. The depletion of TAM by CSF-1R blockade showed increased infiltration of CD8+ cytotoxic T cells and improved treatment response in murine models of breast, prostate, and cervical tumors (123–125). Inhibition of the CSF-1/CSF-1R axis, using antibodies (AMG 820, IMC-CS4) and small molecule inhibitors like pexidartinib, is presently being explored in phase I/II clinical trials (**Table 4**).

**Table 4:** Clinical trials targeting TAM through CSF-1 inhibition.

Cancer type	Trial number	Target	Mechanism of action	Treatment/Intervention
PC, CRC, NSCLC	NCT02713529	CSF1R	CSF1R antibody inhibiting binding of CSF1 and IL34	AMG 820 + Pembrolizumab
Solid tumors	NCT01444404			AMG 820 monotherapy
Advanced solid tumors	NCT02734433	CSF1R, c-KIT, FLT3	Multi-targeted receptor tyrosine kinase inhibitor	Pexidartinib monotherapy
	NCT01525602			Pexidartinib + Paclitaxel
Acral and mucosal melanoma	NCT02071940			Pexidartinib monotherapy
PVNS, GCT-TS, TGCT	NCT02371369			Pexidartinib monotherapy
Sarcoma and Malignant Peripheral Nerve Sheath Tumors	NCT02584647			Pexidartinib + Sirolimus (rapamycin)
Metastatic breast cancer	NCT01596751			Pexidartinib + Eribulin
Breast cancer, neoplasms, and angiosarcoma	NCT01042379			Standard/neoadjuvant therapies with novel agents (Pexidartinib in one arm)
Leukemia and solid tumors	NCT02390752			Pexidartinib monotherapy
Prostate cancer	NCT02472275			Pexidartinib + radiation + antiandrogen therapy
Glioblastoma	NCT01790503			Pexidartinib + radiation + Temozolomide
Metastatic/Advanced PC and CRC	NCT02777710			Pexidartinib + Durvalumab
Melanoma, NSCLC, GIST, HNSCC, and ovarian cancer	NCT02452424			Pexidartinib + Pembrolizumab
Advanced solid tumors	NCT01346358	CSF1R	Monoclonal antibody against CSF1R	IMC-CS4 monotherapy
	NCT02718911			IMC-CS4 + Durvalumab/Tremelimumab
PC	NCT03153410			IMC-CS4 + Cyclophosphamide + Pembrolizumab + GVAX
Breast/Prostate cancer	NCT02265536			IMC-CS4 monotherapy
Metastatic sarcomas	NCT04242238	Switch pocket of CSF1R	Highly selective kinase inhibitor	DCC-3014 + Avelumab
TGCT and advanced tumors	NCT03069469			DCC-3014

PC, pancreatic cancer; CRC, colorectal cancer; PVNS, Pigmented villonodular synovitis; GCT-TS, Giant cell tumors of the tendon sheath; TGCT, Tenosynovial Giant Cell Tumor; CSF1, colony stimulating factor 1; IL-34, interleukin 34; c-KIT, KIT proto-oncogene receptor tyrosine kinase; CSF1R, CSF1 receptor; FLT-3, FMS like tyrosine kinase 3; NSCLC, non-small-cell lung cancer; GIST, gastrointestinal stromal tumor; HNSCC, head and neck squamous cell carcinoma; GVAX, granulocyte-macrophage colony-stimulating factor (GM-CSF) gene-transfected tumor cell vaccine.

Additionally, the plasticity of macrophages opens up new avenues for reprogramming TAM to switch to an anti-tumor, M1-subtype. While drugs targeting toll-like receptors (imiquimod) are already approved for use, many novel antibodies and fusion proteins targeting CD47/SIRPα axis are under investigation (**Table 5**) (126). Adding to that list, some preclinical trials are currently investigating the use of CAR-T adoptive cell transfer and mRNA tumor vaccines. Theoretically, strategies to reprogram TAM by the delivery of mRNA are attractive, but this research is still in its nascent stages.

**Table 5:** Clinical trials investigating reprogramming of TAM in combination with other therapies.

Cancer type	Trial number	Target	Mechanism of action	Treatment/Intervention
Ovarian cancer	NCT03558139	CD47	Monoclonal antibody recognizes CD47 and blocks the “don’t eat me” signal on SIRPα receptor on TAM	Magrolimab + Avelumab
Hodgkin lymphoma	NCT04788043			Magrolimab + Pembrolizumab
Urothelial carcinoma	NCT03869190			Several treatment combinations including Magrolimab
AML	NCT04435691			Magrolimab + Azacitidine + Venetoclax
AML and myelodysplastic syndrome	NCT03248479			Magrolimab +/- Azacitidine
Solid tumors and advanced CRC	NCT02953782			Magrolimab + Cetuximab
Non-Hodgkin lymphoma	NCT02953509			Magrolimab + Rituximab + Gemcitabine + Oxaliplatin
Hematologic malignancies and solid tumors	NCT02663518	CD47	TTI-621 is SIRPαFc, a recombinant fusion protein blocking CD47:SIRPα axis	TTI-621 alone/+ Rituximab/+ Nivolumab
Lymphoma and myeloma	NCT03530683	CD47	SIRPα-IgG4Fc, a recombinant fusion protein binding to CD47	TTI-622 alone/+ Rituximab/+ Nivolumab/+ Carfilzomib
Hematologic cancers and advanced solid tumors	NCT03512340	CD47	Anti-CD47 antibody	SRF231
PDAC	NCT01456585	CD40	CP-870,893 is a fully human, CD40-specific agonist monoclonal antibody	CP-870,893 + Gemcitabine
Metastatic melanoma	NCT01103635			CP-870,893 + Tremelimumab
Metastatic CRC	NCT03555149	CD40	Selicrelumab is a human IgG2 agonistic anti-CD40 monoclonal antibody	Several combinations including Selicrelumab
Metastatic PDAC	NCT03193190			Several combinations including Selicrelumab
Locally advanced and metastatic solid tumors	NCT02304393			Selicrelumab + Atezolizumab

AML, acute myeloid leukemia; CRC, colorectal cancer; PDAC, pancreatic ductal adenocarcinoma; CD47, cluster of differentiation protein-47; TAM, tumor associated macrophages; IgG, immunoglobulin G; SIRPα, signal regulatory protein α.

To this end, TAM are a promising therapeutic target and further research will benefit in the development of combinational regimens utilizing multifaceted targeting of the cancers.

## The CAF–TAM Collaboration

Although CAF and TAM can play both supportive and restrictive roles in carcinogenesis and tumor progression, they are emerging as key players in orchestrating cancer-promoting inflammation and their interactions likely increase the malignancy of tumors (127).

Further to the recruitment of monocytes and M2 polarization, recent data have linked CAF and TAM to a reciprocal interplay with cancer cells. The anti-inflammatory and immunosuppressive M2 phenotype facilitates tumor growth and converts healthy fibroblasts into CAF. Activated CAF secrete factors that promote TAM, cancer cell aggressiveness, EMT, and stemness. In return, cancer cell secrete factors that increase CAF activation and reactivity in a complex that involves various interleukins, chemokines, growth factors and proteinases (128).

As the synergistic interaction between TAM and CAF was only recently identified, only a few studies describe their cell-cell interactions. In CRC and oral squamous cell carcinoma, high levels and combined presence of CAF and TAM within the TME was reported as a negative prognostic factor (7, 129). In high-risk neuroblastoma, pro-inflammatory lipid mediators produced by CAF contributed to tumor growth and were accompanied by a high infiltration of CD163^+^ TAM (130).

The CAF secretome seems to regulate the composition of tumor-related inflammation, including the presence, phenotypes, and levels of infiltrating TAM (13). In this case, CAF together with tumor cells shape the environment to which monocytes/macrophages are recruited to promote tumor progression (127, 131). To evaluate the effects of CAF on tumor growth and metastasis, monocytes were co-cultured with colon cancer cells and stimulated with colon cancer-activated CAF. The inducible factors that drove monocyte differentiation into pro-invasive TAM were primarily characterized as CAF-derived GM-CSF and IL-6, and are known to regulate the presence of TAM and promote cancer cell invasion and metastasis. Therefore, in the triple cross-talk between tumor cells, CAF, and TAM, IL-6 and GM-CSF could become important targets for modulating their interaction (95).

In HCC, osteopontin (OPN) was identified as a key molecule involved in cancer-CAF-TAM interactions. OPN is a chemokine-like phosphorylated glycoprotein released by TAM in the TME. The TAM-secreted OPN promotes the secretion of OPN from CAF and leads to increased cancer cell malignancy through upregulation of proliferation, ECM degradation, and migration. Thus, OPN could be a potential new therapeutic target to inhibit cancer-CAF-TAM interactions in HCC (132).

Based on global gene expression profiles in CRC, bioinformatics and immunohistochemistry identified stromal markers that were significantly associated with resistance to therapy, recurrence and poor prognosis. The predictive power of stromal cell genes was higher than the power of tumor cell genes (4). In accordance with and by investigating the four consensus molecular subtypes (CMS) in CRC, the CMS4 tumors were characterized by heavy infiltration of mesenchymal cells and displayed worse recurrence-free survival and overall survival compared with other CMS subtypes. Moreover, the CMS4 tumors showed a clear upregulation of genes controlling EMT, TGF-β signaling, angiogenesis, matrix remodelling, and inflammation (5).

Compared with TAM, and playing a dominant role in the evolution of TME, a higher density of CAF is usually observed in tumors of the gastrointestinal tract, pancreas, lung, and prostate. It is important to mention that TAM are associated with migration and intravasation of tumor cells, CTC formation, and aiding CTC clusters in the peripheral circulation in the patients (73, 133, 134). Despite them being appealing targets, owing to the lack of selectivity, strategies to attack CAF and TAM have resulted in unwanted side-effects and thereby, limited their clinical use (14, 135).

## The Extracellular Matrix

The ECM mainly consists of proteins and glycosaminoglycans that are constantly remodeled by fibroblasts and macrophages in response to environmental changes.

In preclinical trials on breast and lung cancer, it was reported that CAF-produced collagen and CAF-derived FAP transformed the ECM into an environment facilitating the cancer cell motility through a parallel alignment of the collagen fibers that enhanced the direction and speed of the migrating cells (136).

Regular tissue fibroblasts synthesize and release ECM components such as collagen, elastin, fibronectin, and a variety of proteoglycans that combine to form a web of fibers. This network regulates the homeostasis of cells, tissues, and organs and allows the ECM and tumor cells to resist a wide range of chemical and mechanical stress factors (137).

In a solid tumor, the assembly of ECM fibrils is crucial for the barrier formation and exclusion of immune cells and therapeutics. Further, the collagen network in the stroma is key for the maintenance and exchange of fluids and solutes within the tumor.

Elastin, an abundantly expressed protein in the ECM, is secreted by fibroblasts as a precursor protein, tropoelastin, which assembles in the elastic fibers that are rich in crosslinks. The crosslinks render the elastin insoluble and equip the fibers with the ability to withstand repeated distension. Additionally, the elastin fibers are tightly associated with collagen fibrils which are mediated by the cell surface proteoglycans (138).

Fibronectin, also secreted by fibroblasts, binds to the ECM components such as collagen and fibrin and anchors the fibrils to the cell-surface integrin receptors (139).

The tyrosine kinase inhibitor, Imatinib—specific to ABL1, PDGFR, and c-kit—is used to treat hematological malignancies and gastrointestinal stromal tumors. It is found to increase the flow of fluids through the interstitial compartment of the tumor, improving drug delivery, mainly due to a decreased collagen fibril diameter (140, 141).

CAF and TAM produce various enzymes, including matrix metalloproteinases (MMP), fibrinolysin, and cathepsins that degrade ECM components, accelerate local invasion of tumor cells, and facilitate their dissemination (142, 143). Some ECM degradation fragments may even stimulate angiogenesis and migration (144). MMPs are zinc-dependent ECM-remodelling endopeptidases deeply implicated in almost all steps of metastasis. A high MMP expression in the tumor correlates with poor prognosis and increased risk of recurrence (145). The CAF expression of MMP-11 in CRC, MMP-2 and MMP-9 in breast cancer, and MMP-21 in HCC was significantly related to a high risk of tumor recurrence (146–148).

The presence of hypoxia, acidity, increased interstitial pressure, and aberrant vasculature in the TME confer tumor cells with a survival advantage. The environment inhibits the penetration, navigation, and functionality of cytotoxic immune cells in their quest to kill tumor cells (149, 150).

To prevent intracellular acidity, tumor cells express various proton flux regulators, such as H^+^-ATPases, Na^+^/H^+^ exchangers, monocarboxylate transporters, carbonic anhydrases, and Na^+^/HCO_3_ transporters. Proton pump inhibitors are currently being used in clinical trials (151, 152), in combination with therapies targeting carbonic anhydrases: Acetazolamide (carbonic anhydrase inhibitor) and radiotherapy for small cell lung cancer (NCT03467360), carbonic anhydrase IX inhibitor and Gemcitabine (antimetabolite) for PDAC (NCT03450018), and Acetazolamide and Temozolomide (alkylating agent) for malignant glioma of the brain (NCT03011671). Additional clinical trials of therapies that aim to target ECM and ECM-associated molecules are on-going; however, as therapeutics, ECM degrading agents must be used with caution as they may have fundamental consequences on cell and tissue functions, which could ease the metastatic spread instead of inhibiting tumor progression (153).

## CAF and TAM in Immunotherapy and Anti-Angiogenesis

The introduction of monoclonal antibodies targeting inhibitory receptors on immune cells, known as immune checkpoint inhibitors, has been a great breakthrough in oncology, immensely improving the clinical outcomes of several cancers. This therapeutic strategy enhances the efficacy of anti-tumor immune responses and revitalizes exhausted killer cells such as CD8^+^ T cells and NK cells (154).

The exclusion of immune cells from solid tumors is not only caused by the physical and chemical barrier of the ECM, but also by the immune checkpoint ligands expressed by cancer cells, CAF, and TAM (155). In line with this, a study on tissue samples from patients with PDAC demonstrated that PD-L1 and PD-L2 (both ligands to PD-1) expressed by CAF were involved in immune cell exclusion and anergy (156).

Adding to the complexity of stromal cell functions, preclinical studies suggest that some CAF, along with normal fibroblasts, have the ability to overrule oncogenic signaling from the surroundings and act as tumor suppressors (20, 157). Whether these fibroblasts are subtypes of normal fibroblasts resistant to CAF conversion or distinct anti-tumor CAF subpopulations remains unknown. However, the CAF/TAM collaboration do play a vital tumor-promoting role. It fuels the growth of tumors; induces stemness and EMT in cancer cells by the production of cytokines, chemokines, e.g. interleukins, TGF-β, CCL, and CXCL chemokines (158). It supplies the tumors with energy-rich metabolites and upregulate the tumor-cell mitochondrial oxidative phosphorylation (159). Thus, therapeutic regimens targeting the TAM-CAF interaction in combination with immunotherapy could improve anti-tumor therapeutic efficacy (160).

CSF-1R receptors are overexpressed on TAM in many cancers, controlling the production, differentiation, and function of macrophages. In a mouse model, a CSF-1R-inhibitor blocked the production of inflammatory mediators in TAM, inhibited the recruitment of bone marrow-derived suppressor cells (BMDSC), and enhanced T-cell infiltration and CD8^+^ T cell activity. However, the inhibition of CSF-1R signaling caused CAF to secrete chemokines and chemokine ligands that neutralized the CSF-1R inhibitor. The supplementation of a chemokine receptor antagonist reduced the tumor burden, and tumor growth was completely blocked when an immune checkpoint inhibitor (anti-PD-1) was further added to the combination (161).

There are currently several clinical trials evaluating the effect of CSF1R monoclonal antibodies in combination with immune checkpoint inhibitors in a variety of solid tumors (**Table 4**).

In a human trial on solid tumors, dual antibody blockade (anti-TGF-β and anti-PD-L1) led to a significant increase in the number of cytotoxic CD8^+^ T cells in the TME. The co-inhibition of TGF-β and PD-L1 converted an immune excluded tumor phenotype to an inflamed phenotype, supporting the fact that TGF-β signaling prevents T-cell invasion. T cell localization was not affected with either antibody as monotherapy (162). Thus, TAM expressing immune checkpoint receptor ligands limit the functions of effector T cells, NK cells, and dendritic cells, and attenuate the effects of immune checkpoint inhibitor therapy (101, 102).

To prevent phagocytosis, upregulated CD47 surface proteins on tumor cells provide a “do not eat me” signal by ligating the inhibitory TAM-receptor signal regulatory protein alpha (SIRPα). As CD47 also promotes the proliferation of cancer cells *via* the PI3K/AKT pathway, the CD47 signaling pathway is considered an important mechanism of therapy resistance. Inhibition of CD47 could be a promising therapeutic strategy, particularly in combination with immune checkpoint inhibitors.

In mouse models of melanoma, colon carcinoma, and lymphoma, dual targeting of CD47 and PD-L1 was found to enhance anti-tumor effects (163–165) and several clinical trials evaluating the efficacy of CD47 or SIRPα monoclonal antibodies as monotherapy or in combination with immune checkpoint inhibitors are underway (**Table 5**; ClinicalTrials.gov).

As VEGF-A is overexpressed in both tumor cells, CAF, and TAM and is associated with cancer progression and dissemination, it represents the main target of anti-angiogenic drugs in cancer therapy. These drugs are widely used in the treatment of various cancers and have resulted in increased overall survival or progression-free survival in gynecologic cancers (166), CRC (167), and gastric cancer (168). However, due to antiangiogenic drug resistance of tumor cells, metastasis and mortality continue to occur during and after cessation of treatment. This resistance comprises the amplification of pro-angiogenic genes, secretion of multiple proangiogenic factors, and recruitment of proangiogenic BMDSC (169). Bevacizumab, a humanized monoclonal antibody that targets all VEGF-A isoforms and the first anti-angiogenic drug approved for clinical application, is efficacious in various malignancies such as CRC and glioblastoma (170). Today, most clinical studies use anti-angiogenetic drugs in combinatory regimens, e.g. lenvatinib (multiple kinase inhibitor) inhibiting both VEGFR 1–3 and PDGFR and Pembrolizumab (anti-PD-1) for the treatment of endometrial cancer (NCT03517449).

The anti-diabetic drug metformin appears to be a promising therapeutic agent in neoadjuvant and adjuvant settings. The metformin‐induced antitumor and anti‐angiogenic effects are partly related to the skewing of TAM polarization from M2‐ to M1‐like phenotype and significant inhibition of tumor angiogenesis. Currently, there is very little insight into the mechanism through which metformin modulates macrophage function. However, an *in vitro* study on breast cancer cells and TAM polarization revealed that metformin treatment activated AMPK-NF-κB signaling in cancer cells. These molecules participate in the regulation of M1 and M2 inducing cytokines. Metformin was observed to increase macrophage expression of M1-related cytokines IL-12 and TNF-α and attenuate the expression of the M2-related cytokines IL-8, IL-10, and TGF-β. Furthermore, the secretion of important cytokines for the M2 phenotype (e.g. IL-4, IL-10, and IL-13) was inhibited in metformin-treated cancer cells (171).

In cultures of human cholangiocarcinoma cells, and at concentrations corresponding to plasma levels of metformin in diabetic patients, metformin inhibited proliferation and cell migration and induced apoptosis. Expression of vimentin (mesenchymal marker) and EMT genes was downregulated and expression of cytokeratin-19 (epithelial marker) was upregulated (172). The findings from the multiple ongoing trials (173) may convey a deeper understanding of the anti-tumor function of metformin in the near future.

## Discussion

**Figure 3:**
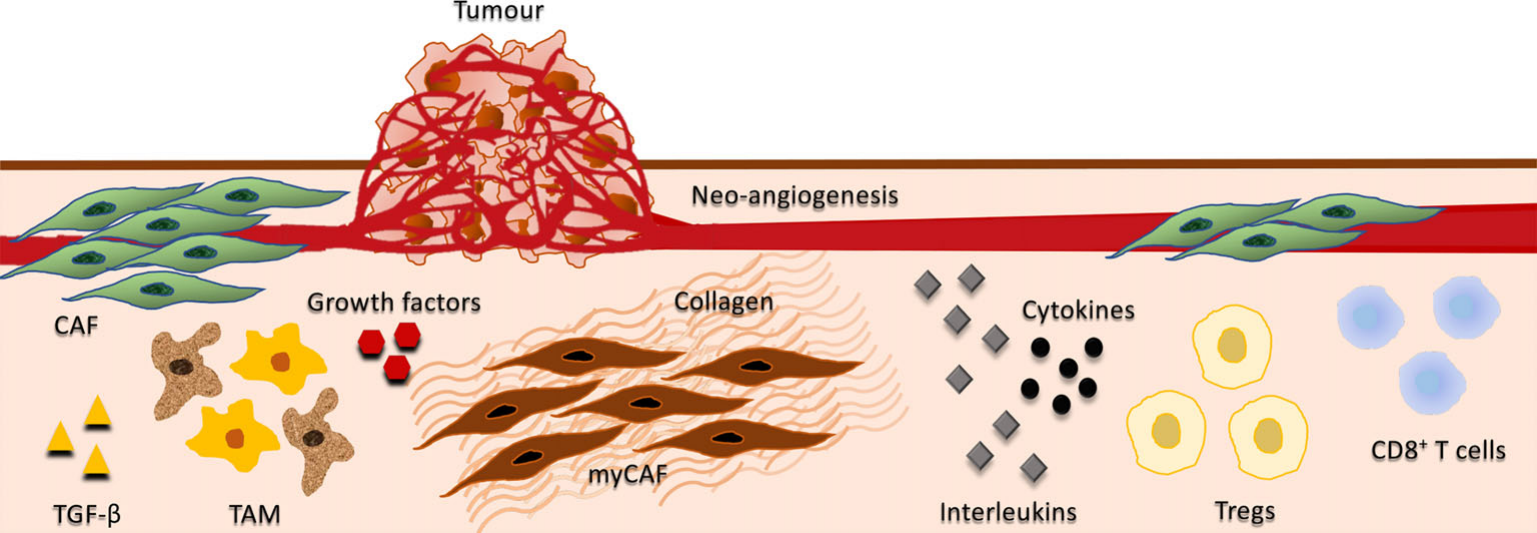
Interplay between tumor, stromal, and immune cells. Depicts the interplay between cancer-associated fibroblasts (CAF), tumor-associated macrophages (TAM), growth factors, cytokines, interleukins, and immune cells in the tumor microenvironment (TME). CAF are the predominant cell type in the tumor stroma, contributing to the proliferative, pro-inflammatory, immunosuppressive, angiogenic, pro-invasive and pro-metastatic TME. They secrete various growth factors including TGF-β, vascular endothelial growth factor (VEGF), fibroblast growth factor 5, growth differentiation factor 15, and hepatocyte growth factor. CAF also produce cytokines and interleukins that may have both immunosuppressive and immuno-activating effects on various leukocytes, including CD8^+^ T cells, immunosuppressive regulatory T cells (T_regs_) and macrophages. A subset of CAFs have myofibroblasts characteristics (myCAF) and play a major role in the development of the fibrotic stroma in the TME including the regulation of collagen fibre elongation. Growth factors and immunosuppressive cytokines produced by TAM enhance motility, intravasation, and invasion of tumor cells, while stimulating angiogenesis and suppressing T cell infiltration. Additionally, TGF‐β produced by TAMs activate immunosuppressive T_regs_.

In a solid tumor, the balance between growth and differentiation is determined by the TME. TAM and CAF promote cancer evolution through the inflammatory, immunosuppressive, angiogenic, energy-rich environment, and also suppress cancer cells *via* predominantly unknown mechanisms. The presence and precise functions of CAF and TAM in the TME are extremely complex (**Figure 3**) and incompletely understood, and only a few studies describe the interplay between these cells. The general perception is that the TME strongly modulates tumor cells through all phases of disease progression, and as each tumor is comprised of multiple clones with myriads of cell types and signaling molecules, the heterogeneity of each tumor may therefore require unique therapeutic approaches.

Improving our understanding of the TME including the impact of stromal cells, immune cells, and ECM components, is vital for the innovation of therapeutic strategies. Hence, cell-cell communication within the TME should be integrated into future cancer research. However, the manipulation of the immune system and/or stromal components within the TME during cancer treatment can be unpredictable. The regulation/eradication of α-SMA^+^ or FAP^+^ CAF have had variable results and currently, targeting CAF or TAM individually does not seem to be an appropriate approach.

A CAF-directed therapy could be designed against specific pro-tumorigenic factors that in turn could prevent CAF activation or CAF functions. The reprogramming of CAF back into a normal resting phenotype would be a desirable option; however, targeting FAP has had a minimal response in human trials (20). Drugs that target CAF may emerge as a complement to immunotherapies in solid tumors, though a major obstacle in the precision strategy of CAF-based therapy is that neither α-SMA nor FAP is exclusively expressed by CAF.

In theory, TAM antagonists could be used to overcome resistance to immunotherapy; nevertheless, the type of approach is yet to be determined. The lack of macrophage selectivity has so far hindered its introduction into the clinic.

Monoclonal antibodies blocking the interaction between CD47 on tumor cells and SIRPα on innate immune cells is another interesting direction for future research. Other potential treatment targets are the MMPs. In the TME, MMPs are expressed by various cell types, and a number of MMP inhibitors have been tested in phase 1, 2, and 3 clinical trials. Unfortunately, all trials across different cancer types and stages have failed to provide any improvements in the clinical outcomes (174). Nonetheless, the field is advancing fast with the development of small-molecule inhibitors and antibodies targeting specific domains of pro-tumorigenic MMPs.

In PDAC, the TME is an important contributor to tumor progression and prognosis. The increasing amount of ECM and fibrosis promote tumor progression and correlate with shorter survival. The aberrant TGF-β signaling in cancer cells leads to an increased epithelial signal transducer and activator of transcription 3 (STAT3) activity, resulting in increased ECM fibrosis (175). Therefore, the concept of reducing tumor aggressiveness by interfering with STAT3 hyperactivity seems intriguing.

Notably, a recent study demonstrated that increased phosphorylation of STAT3 in CAF was associated with reduced overall survival in CRC patients (176). To improve response rates and increase the number of responding cancer types, combination therapies using STAT3 inhibitors and immune checkpoint inhibitors are now being undertaken (177).

In conclusion, combinations of immune-modulating agents are gaining more and more ground in oncology. CAF and TAM hold significant potential to improve targeted therapy and outcomes in cancer treatment when combined with existing therapies. Although in its naive stages, the TME modulating technology is an active field of research that holds immense prospects for researchers, clinicians, and patients.

## Author Contributions

HR: idea, design, intelectual contents, writing the manuscript, and crreation of ilustration. AO: intelectual contents, writing the manuscript, and creation of illustrations. SG: language editing, intelectual contents, manuscript structure, and creation of tables. IG: overall design, intelectual contents, and editing. All authors contributed to the article and approved the submitted version.

## Conflict of Interest

The authors declare that the research was conducted in the absence of any commercial or financial relationships that could be construed as a potential conflict of interest.

## Abbreviations

α-SMA, alpha smooth muscle actin; b-FGF, basic fibroblast growth factor; CAF, cancer-associated fibroblasts; CAFEx, CAF-derived exosomes; CCL, C-C motif ligand; CRC, colorectal cancer; CTC, circulating tumor cell; CXCL, C-X-C motif ligand; CCR, C-C chemokine receptor; CD, cluster of differentiation; CSF1R, colony-stimulating factor 1 receptor; CSM, consensus molecular subtypes; ECM, extracellular matrix; EGF, epidermal growth factor; EMT, epithelial-mesenchymal transition; FAP, fibroblast activation protein; GM-CSF, granulocyte-macrophage colony-stimulating factor; HCC, hepatocellular carcinoma; IL, interleukin; myCAF, cancer-associated myofibroblast; miR, micro-RNA; MMP, matrix metalloproteinase; OPN, osteopontin; PDAC, pancreatic ductal adenocarcinoma; PD-1, programmed death receptor 1; PD-L1 and 2, programmed death receptor ligand 1 and 2; PDGF, platelet-derived growth factor; PDGFR, platelet-derived growth receptor; STAT3, signal transducer and activator of transcription 3; TAM, tumor-associated macrophages; TGF, transforming growth factor; TME, tumor microenvironment; TNF, tumor necrosis factor; TSE, tumor-derived exosomes; TSF, tumor-derived factors; VEGF, vascular endothelial growth factor; VEGFR, vascular endothelial growth factor receptor.

## References

[1] Hanahan D Weinberg R. Hallmarks of Cancer: The Next Generation. Cell Press (2011) 144:646–74. 10.1016/j.cell.2011.02.013 21376230

[2] Gentles AJ Newman AM Liu CL Bratman SV Feng W Kim D. The Prognostic Landscape of Genes and Infiltrating Immune Cells Across Human Cancers. Nat Med (2015) 21:938–45. 10.1038/nm.3909 PMC4852857 26193342

[3] Hashimoto O Yoshida M Koma Y-I Yanai T Hasegawa D Kosaka Y. Collaboration of Cancer-Associated Fibroblasts and Tumour-Associated Macrophages for Neuroblastoma Development. J Pathol (2016) 240:211–23. 10.1002/path.4769 PMC5095779 27425378

[4] Calon A Lonardo E Berenguer-Llergo A Espinet E Hernando-Momblona X Iglesias M. Stromal Gene Expression Defines Poor-Prognosis Subtypes in Colorectal Cancer. Nat Genet (2015) 47:320–9. 10.1038/ng.3225 25706628

[5] Guinney J Dienstmann R Wang X de Reyniès A Schlicker A Soneson C. The Consensus Molecular Subtypes of Colorectal Cancer. Nat Med (2015) 21:1350–6. 10.1038/nm.3967 PMC4636487 26457759

[6] Zhang Q Liu L Gong C Shi H Zeng Y Wang X. Prognostic Significance of Tumor-Associated Macrophages in Solid Tumor: A Meta-Analysis of the Literature. PloS One (2012) 7:e50946. 10.1371/journal.pone.0050946 23284651 PMC3532403

[7] Herrera M Herrera A Domínguez G Silva J García V García JM. Cancer-Associated Fibroblast and M2 Macrophage Markers Together Predict Outcome in Colorectal Cancer Patients. Cancer Sci (2013) 104:437–44. 10.1111/cas.12096 PMC7657228 23298232

[8] Roma-Rodrigues C Mendes R Baptista PV Fernandes AR. Targeting Tumor Microenvironment for Cancer Therapy. Int J Mol Sci (2019) 20:840. 10.3390/ijms20040840 PMC6413095 30781344

[9] Vallée A Lecarpentier Y. Tgf-β in Fibrosis by Acting as a Conductor for Contractile Properties of Myofibroblasts. Cell Biosci (2019) 9:98. 10.1186/s13578-019-0362-3 31827764 PMC6902440

[10] Neuzillet C Tijeras-Raballand A Ragulan C Cros J Patil Y Martinet M. Inter- and Intra-Tumoural Heterogeneity in Cancer-Associated Fibroblasts of Human Pancreatic Ductal Adenocarcinoma. J Pathol (2019) 248:51–65. 10.1002/path.5224 30575030 PMC6492001

[11] Tauriello DVF Palomo-Ponce S Stork D Berenguer-Llergo A Badia-Ramentol J Iglesias M. Tgfβ Drives Immune Evasion in Genetically Reconstituted Colon Cancer Metastasis. Nature (2018) 554:538–43. 10.1038/nature25492 29443964

[12] Liu T Han C Wang S Fang P Ma Z Xu L. Cancer-Associated Fibroblasts: An Emerging Target of Anti-Cancer Immunotherapy. J Hematol Oncol (2019) 12:86. 10.1186/s13045-019-0770-1 31462327 PMC6714445

[13] Kalluri R. The Biology and Function of Fibroblasts in Cancer. Nat Rev Cancer (2016) 16:582—598. 10.1038/nrc.2016.73 27550820

[14] Sahai E Astsaturov I Cukierman E DeNardo DG Egeblad M Evans RM. A Framework for Advancing Our Understanding of Cancer-Associated Fibroblasts. Nat Rev Cancer (2020) 20:174–86. 10.1038/s41568-019-0238-1 PMC7046529 31980749

[15] Öhlund D Elyada E Tuveson D. Fibroblast Heterogeneity in the Cancer Wound. J Exp Med (2014) 211:1503–23. 10.1084/jem.20140692 PMC4113948 25071162

[16] Raskov H Orhan A Salanti A Gögenur I. Premetastatic Niches, Exosomes and Circulating Tumor Cells: Early Mechanisms of Tumor Dissemination and the Relation to Surgery. Int J Cancer (2020) 146:3244–55. 10.1002/ijc.32820 31808150

[17] Albrengues J Bertero T Grasset E Bonan S Maiel M Bourget I. Epigenetic Switch Drives the Conversion of Fibroblasts Into Proinvasive Cancer-Associated Fibroblasts. Nat Commun (2015) 6:10204. 10.1038/ncomms10204 26667266 PMC4682161

[18] Sanz-Moreno V Gaggioli C Yeo M Albrengues J Wallberg F Viros A. ROCK and JAK1 Signaling Cooperate to Control Actomyosin Contractility in Tumor Cells and Stroma. Cancer Cell (2011) 20:229–45. 10.1016/j.ccr.2011.06.018 21840487

[19] Bhowmick NA Neilson EG Moses HL. Stromal Fibroblasts in Cancer Initiation and Progression. Nature (2004) 432:332–7. 10.1038/nature03096 PMC3050735 15549095

[20] Gieniec KA Butler LM Worthley DL Woods SL. Cancer-Associated Fibroblasts-Heroes or Villains? Br J Cancer (2019) 121:293–302. 10.1038/s41416-019-0509-3 31289350 PMC6738083

[21] Yoshida GJ. Regulation of Heterogeneous Cancer-Associated Fibroblasts: The Molecular Pathology of Activated Signaling Pathways. J Exp Clin Cancer Res (2020) 39:112. 10.1186/s13046-020-01611-0 32546182 PMC7296768

[22] Kieffer Y Hocine HR Gentric G Pelon F Bernard C Bourachot B. Single-Cell Analysis Reveals Fibroblast Clusters Linked to Immunotherapy Resistance in Cancer. Cancer Discovery (2020) 10:1330–51. 10.1158/2159-8290.CD-19-1384 32434947

[23] Gerling M Büller N Kirn L Joost S Frings O Englert B. Stromal Hedgehog Signalling is Downregulated in Colon Cancer and its Restoration Restrains Tumour Growth. Nat Commun (2016) 5:12321. 10.1038/ncomms12936 PMC4980446 27492255

[24] Öhlund D Handly-Santana A Biffi G Elyada E Almeida AS Ponz-Sarvise M. Distinct Populations of Inflammatory Fibroblasts and Myofibroblasts in Pancreatic Cancer. J Exp Med (2017) 214:579–96. 10.1084/jem.20162024 PMC5339682 28232471

[25] Shin K Lim A Zhao C Sahoo D Pan Y Spiekerkoetter E. Hedgehog Signaling Restrains Bladder Cancer Progression by Eliciting Stromal Production of Urothelial Differentiation Factors. Cancer Cell (2014) 26:521–33. 10.1016/j.ccell.2014.09.001 PMC4326077 25314078

[26] Özdemir BC Pentcheva-Hoang T Carstens JL Zheng X Wu C-C Simpson TR. Depletion of Carcinoma-Associated Fibroblasts and Fibrosis Induces Immunosuppression and Accelerates Pancreas Cancer With Reduced Survival. Cancer Cell (2015) 28:831—833. 10.1016/j.ccell.2015.11.002 28843279

[27] Rhim AD Oberstein PE Thomas DH Mirek ET Palermo CF Sastra SA. Stromal Elements Act to Restrain, Rather Than Support, Pancreatic Ductal Adenocarcinoma. Cancer Cell (2014) 25:735–47. 10.1016/j.ccr.2014.04.021 PMC4096698 24856585

[28] Arina A Idel C Hyjek EM Alegre M-L Wang Y Bindokas VP. Tumor-Associated Fibroblasts Predominantly Come From Local and Not Circulating Precursors. Proc Natl Acad Sci USA (2016) 113:7551–6. 10.1073/pnas.1600363113 PMC4941507 27317748

[29] Kretzschmar K Weber C Driskell RR Calonje E Watt FM. Compartmentalized Epidermal Activation of β-Catenin Differentially Affects Lineage Reprogramming and Underlies Tumor Heterogeneity. Cell Rep (2016) 14:269–81. 10.1016/j.celrep.2015.12.041 PMC4713864 26771241

[30] Elyada E Bolisetty M Laise P Flynn WF Courtois ET Burkhart RA. Cross-Species Single-Cell Analysis of Pancreatic Ductal Adenocarcinoma Reveals Antigen-Presenting Cancer-Associated Fibroblasts. Cancer Discovery (2019) 9:1102–23. 10.1158/2159-8290.CD-19-0094 PMC6727976 31197017

[31] Duda D Duyverman A Kohno M Snuderl M Steller E Fukumura D. Malignant Cells Facilitate Lung Metastasis by Bringing Their Own Soil. Proc Natl Acad Sci USA (2010) 107:21677–82. 10.1073/pnas.1016234107 PMC3003109 21098274

[32] Kraman M Bambrough PJ Arnold JN Roberts EW Magiera L Jones JO. Suppression of Antitumor Immunity by Stromal Cells Expressing Fibroblast Activation Protein-Alpha. Science (2010) 330:827–30. 10.1126/science.1195300 21051638

[33] Lo A Li C-P Buza EL Blomberg R Govindaraju P Avery D. Fibroblast Activation Protein Augments Progression and Metastasis of Pancreatic Ductal Adenocarcinoma. JCI Insight (2017) 2:1–11. 10.1172/jci.insight.92232 PMC5841864 28978805

[34] Fearon DT. The Carcinoma-Associated Fibroblast Expressing Fibroblast Activation Protein and Escape From Immune Surveillance. Cancer Immunol Res (2014) 2:187–93. 10.1158/2326-6066.CIR-14-0002 24778314

[35] Feig C Jones JO Kraman M Wells RJB Deonarine A Chan DS. Targeting CXCL12 From FAP-expressing Carcinoma-Associated Fibroblasts Synergizes With anti-PD-L1 Immunotherapy in Pancreatic Cancer. Proc Natl Acad Sci USA (2013) 110:20212–7. 10.1073/pnas.1320318110 PMC3864274 24277834

[36] Costa A Kieffer Y Scholer-Dahirel A Pelon F Bourachot B Cardon M. Fibroblast Heterogeneity and Immunosuppressive Environment in Human Breast Cancer. Cancer Cell (2018) 33:463+. 10.1016/j.ccell.2018.01.011 29455927

[37] Amakye D Jagani Z Dorsch M. Unraveling the Therapeutic Potential of the Hedgehog Pathway in Cancer. Nat Med (2013) 19:1410–22. 10.1038/nm.3389 24202394

[38] Gunderson AJ Yamazaki T McCarty K Phillips M Alice A Bambina S. Blockade of Fibroblast Activation Protein in Combination With Radiation Treatment in Murine Models of Pancreatic Adenocarcinoma. PloS One (2019) 14:e0211117. 10.1371/journal.pone.0211117 30726287 PMC6364920

[39] Loeffler M Krüger JA Niethammer AG Reisfeld RA. Targeting Tumor-Associated Fibroblasts Improves Cancer Chemotherapy by Increasing Intratumoral Drug Uptake. J Clin Invest (2006) 116:1955–62. 10.1172/JCI26532 PMC1481657 16794736

[40] Lo A Wang L-CS Scholler J Monslow J Avery D Newick K. Tumor-Promoting Desmoplasia is Disrupted by Depleting Fap-Expressing Stromal Cells. Cancer Res (2015) 75:2800–10. 10.1158/0008-5472.CAN-14-3041 PMC4506263 25979873

[41] Gandellini P Andriani F Merlino G D’Aiuto F Roz L Callari M. Complexity in the Tumour Microenvironment: Cancer Associated Fibroblast Gene Expression Patterns Identify Both Common and Unique Features of Tumour-Stroma Crosstalk Across Cancer Types. Semin Cancer Biol (2015) 35:96–106. 10.1016/j.semcancer.2015.08.008 26320408

[42] Dominguez CX Müller S Keerthivasan S Koeppen H Hung J Gierke S. Single-Cell RNA Sequencing Reveals Stromal Evolution Into LRRC15^+^ Myofibroblasts as a Determinant of Patient Response to Cancer Immunotherapy. Cancer Discovery (2020) 10:232–53. 10.1158/2159-8290.CD-19-0644 31699795

[43] Shi Y Gao W Lytle NK Huang P Yuan X Dann AM. Targeting LIF-mediated Paracrine Interaction for Pancreatic Cancer Therapy and Monitoring. Nature (2019) 569:131–5. 10.1038/s41586-019-1130-6 PMC6565370 30996350

[44] Cazet AS Hui MN Elsworth BL Wu SZ Roden D Chan C-L. Targeting Stromal Remodeling and Cancer Stem Cell Plasticity Overcomes Chemoresistance in Triple Negative Breast Cancer. Nat Commun (2018) 9:2897. 10.1038/s41467-018-05220-6 30042390 PMC6057940

[45] Chen W-J Ho C-C Chang Y-L Chen H-Y Lin C-A Ling T-Y. Cancer-Associated Fibroblasts Regulate the Plasticity of Lung Cancer Stemness *Via* Paracrine Signalling. Nat Commun (2014) 5:3472. 10.1038/ncomms4472 24668028

[46] Iliopoulos D Hirsch HA Wang G Struhl K. Inducible Formation of Breast Cancer Stem Cells and Their Dynamic Equilibrium With non-Stem Cancer Cells *Via* IL6 Secretion. Proc Natl Acad Sci USA (2011) 108:1397–402. 10.1073/pnas.1018898108 PMC3029760 21220315

[47] Tsuyada A Chow A Wu J Somlo G Chu P Loera S. CCL2 Mediates Cross-Talk Between Cancer Cells and Stromal Fibroblasts That Regulates Breast Cancer Stem Cells. Cancer Res (2012) 72:2768–79. 10.1158/0008-5472.CAN-11-3567 PMC3367125 22472119

[48] Jia C Wang G Wang T Fu B Zhang Y Huang L. Cancer-Associated Fibroblasts Induce Epithelial-Mesenchymal Transition *Via* the Transglutaminase 2-Dependent IL-6/IL6R/STAT3 Axis in Hepatocellular Carcinoma. Int J Biol Sci (2020) 16:2542–58. 10.7150/ijbs.45446 PMC7415430 32792856

[49] Goulet CR Champagne A Bernard G Vandal D Chabaud S Pouliot F. Cancer-Associated Fibroblasts Induce Epithelial–Mesenchymal Transition of Bladder Cancer Cells Through Paracrine IL-6 Signalling. BMC Cancer (2019) 19:137. 10.1186/s12885-019-5353-6 30744595 PMC6371428

[50] Rajagopal C Harikumar KB. The Origin and Functions of Exosomes in Cancer. Front Oncol (2018) 8:66. 10.3389/fonc.2018.00066 29616188 PMC5869252

[51] Rai A Greening DW Chen M Xu R Ji H Simpson RJ. Exosomes Derived From Human Primary and Metastatic Colorectal Cancer Cells Contribute to Functional Heterogeneity of Activated Fibroblasts by Reprogramming Their Proteome. Proteomics (2019) 19:1800148. 10.1002/pmic.201800148 30582284

[52] Wang J Yang K Yuan W Gao Z. Determination of Serum Exosomal H19 as a Noninvasive Biomarker for Bladder Cancer Diagnosis and Prognosis. Med Sci Monit Int Med J Exp Clin Res (2018) 24:9307–16. 10.12659/MSM.912018 PMC6320644 30576305

[53] Li M Zhou Y Xia T Zhou X Huang Z Zhang H. Circulating microRNAs From the miR-106a-363 Cluster on Chromosome X as Novel Diagnostic Biomarkers for Breast Cancer. Breast Cancer Res Treat (2018) 170:257–70. 10.1007/s10549-018-4757-3 PMC5999170 29557526

[54] Liu Q Yu Z Yuan S Xie W Li C Hu Z. Circulating Exosomal microRNAs as Prognostic Biomarkers for non-Small-Cell Lung Cancer. Oncotarget (2017) 8:13048–58. 10.18632/oncotarget.14369 PMC5355076 28055956

[55] Sandfeld-Paulsen B Jakobsen KR Bæk R Folkersen BH Rasmussen TR Meldgaard P. Exosomal Proteins as Diagnostic Biomarkers in Lung Cancer. J Thorac Oncol (2016) 11:1701–10. 10.1016/j.jtho.2016.05.034 27343445

[56] Liu W Hu J Zhou K Chen F Wang Z Liao B. Serum Exosomal miR-125b is a Novel Prognostic Marker for Hepatocellular Carcinoma. Onco Targets Ther (2017) 10:3843–51. 10.2147/OTT.S140062 PMC5546809 28814883

[57] Tokuhisa M Ichikawa Y Kosaka N Ochiya T Yashiro M Hirakawa K. Exosomal miRNAs From Peritoneum Lavage Fluid as Potential Prognostic Biomarkers of Peritoneal Metastasis in Gastric Cancer. PloS One (2015) 10:e0130472–e0130472. 10.1371/journal.pone.0130472 26208314 PMC4514651

[58] Shao N Xue L Wang R Luo K Zhi F Lan Q. Mir-454-3p Is an Exosomal Biomarker and Functions as a Tumor Suppressor in Glioma. Mol Cancer Ther (2019) 18:459–69. 10.1158/1535-7163.MCT-18-0725 30413650

[59] Zeng Z Li Y Pan Y Lan X Song F Sun J. Cancer-Derived Exosomal miR-25-3p Promotes Pre-Metastatic Niche Formation by Inducing Vascular Permeability and Angiogenesis. Nat Commun (2018) 9:5395. 10.1038/s41467-018-07810-w 30568162 PMC6300604

[60] Kong J Tian H Zhang F Zhang Z Li J Liu X. Extracellular Vesicles of Carcinoma-Associated Fibroblasts Creates a Pre-Metastatic Niche in the Lung Through Activating Fibroblasts. Mol Cancer (2019) 18:175. 10.1186/s12943-019-1101-4 31796058 PMC6892147

[61] Ortiz-Otero N Clinch AB Hope J Wang W Reinhart-King CA King MR. Cancer Associated Fibroblasts Confer Shear Resistance to Circulating Tumor Cells During Prostate Cancer Metastatic Progression. Oncotarget (2020) 11(12):1037–50. 10.18632/oncotarget.27510 PMC7105166 32256977

[62] Hoshino A Costa-Silva B Shen T Rodrigues G Hashimoto A Tesic Mark M. Tumour Exosome Integrins Determine Organotropic Metastasis. Nature (2015) 527:329–35. 10.1038/nature15756 PMC4788391 26524530

[63] Cluntun AA Lukey MJ Cerione RA Locasale JW. Glutamine Metabolism in Cancer: Understanding the Heterogeneity. Trends Cancer (2017) 3:169–80. 10.1016/j.trecan.2017.01.005 PMC5383348 28393116

[64] Yan W Wu X Zhou W Fong MY Cao M Liu J. Cancer-Cell-Secreted Exosomal miR-105 Promotes Tumour Growth Through the MYC-dependent Metabolic Reprogramming of Stromal Cells. Nat Cell Biol (2018) 20:597+. 10.1038/s41556-018-0083-6 29662176 PMC5920728

[65] Bhagat T Von Ahrens D Dawlaty M Zou Y Baddour J Achreja A. Lactate-Mediated Epigenetic Reprogramming Regulates Formation of Human Pancreatic Cancer-Associated Fibroblasts. Elife (2019) 1:e50663. 10.7554/eLife.50663 PMC6874475 31663852

[66] Gong J Lin Y Zhang H Liu C Cheng Z Yang X. Reprogramming of Lipid Metabolism in Cancer-Associated Fibroblasts Potentiates Migration of Colorectal Cancer Cells. Cell Death Dis (2020) 11:267. 10.1038/s41419-020-2434-z 32327627 PMC7181758

[67] Scherz-Shouval R Santagata S Mendillo ML Sholl LM Ben-Aharon I Beck AH. The Reprogramming of Tumor Stroma by HSF1 is a Potent Enabler of Malignancy. Cell (2014) 158:564–78. 10.1016/j.cell.2014.05.045 PMC4249939 25083868

[68] Kasashima H Duran A Martinez-Ordoñez A Nakanishi Y Kinoshita H Linares J. Stromal SOX2 Upregulation Promotes Tumorigenesis Through the Generation of a SFRP1/2-Expressing Cancer-Associated Fibroblast Population. Dev Cell (2021) 56:95–110. 10.1016/j.devcel.2020.10.014 33207226 PMC7856011

[69] Attieh Y Clark AG Grass C Richon S Pocard M Mariani P. Cancer-Associated Fibroblasts Lead Tumor Invasion Through Integrin-β3-Dependent Fibronectin Assembly. J Cell Biol (2017) 216:3509–20. 10.1083/jcb.201702033 PMC5674886 28931556

[70] Olive KP Jacobetz MA Davidson CJ Gopinathan A McIntyre D Honess D. Inhibition of Hedgehog Signaling Enhances Delivery of Chemotherapy in a Mouse Model of Pancreatic Cancer. Sci (80-) (2009) 324:1457–61. 10.1126/science.1171362 PMC2998180 19460966

[71] Ao Z Shah S Machlin L Parajuli R Miller P Rawal S. Identification of Cancer-Associated Fibroblasts in Circulating Blood From Patients With Metastatic Breast Cancer. Cancer Res (2015) 75:4681–7. 10.1158/0008-5472.CAN-15-1633 26471358

[72] Leach J Morton JP Sansom OJ. Neutrophils: Homing in on the Myeloid Mechanisms of Metastasis. Mol Immunol (2019) 110:69–76. 10.1016/j.molimm.2017.12.013 29269005 PMC6544568

[73] Aceto N Bardia A Miyamoto D Donaldson M Wittner B Spencer J. Circulating Tumor Cell Clusters are Oligoclonal Precursors of Breast Cancer Metastasis. Cell (2014) 158:1110–22. 10.1016/j.cell.2014.07.013 PMC4149753 25171411

[74] Szczerba BM Castro-Giner F Vetter M Krol I Gkountela S Landin J. Neutrophils Escort Circulating Tumour Cells to Enable Cell Cycle Progression. Nature (2019) 566:553–7. 10.1038/s41586-019-0915-y 30728496

[75] Aizawa T Karasawa H Funayama R Shirota M Suzuki T Maeda S. Cancer-Associated Fibroblasts Secrete Wnt2 to Promote Cancer Progression in Colorectal Cancer. Cancer Med (2019) 8:6370–82. 10.1002/cam4.2523 PMC6797671 31468733

[76] Lambrechts D Wauters E Boeckx B Aibar S Nittner D Burton O. Phenotype Molding of Stromal Cells in the Lung Tumor Microenvironment. Nat Med (2018) 24:1277–89. 10.1038/s41591-018-0096-5 29988129

[77] Sica A Mantovani A. Macrophage Plasticity and Polarization. Vivo Veritas J Clin Invest (2012) 122:787–95. 10.1172/JCI59643 PMC3287223 22378047

[78] Das A Sinha M Datta S Abas M Chaffee S Sen CK. Monocyte and Macrophage Plasticity in Tissue Repair and Regeneration. Am J Pathol (2015) 185:2596–606. 10.1016/j.ajpath.2015.06.001 PMC4607753 26118749

[79] Hashimoto D Chow A Noizat C Teo P Beasley MB Leboeuf M. Tissue-Resident Macrophages Self-Maintain Locally Throughout Adult Life With Minimal Contribution From Circulating Monocytes. Immunity (2013) 38:792–804. 10.1016/j.immuni.2013.04.004 23601688 PMC3853406

[80] DeNardo DG Ruffell B. Macrophages as Regulators of Tumour Immunity and Immunotherapy. Nat Rev Immunol (2019) 19:369–82. 10.1038/s41577-019-0127-6 PMC7339861 30718830

[81] Beltraminelli T De Palma M. Biology and Therapeutic Targeting of Tumour-Associated Macrophages. J Pathol (2020) 250:573–92. 10.1002/path.5403 32086811

[82] Shapouri-Moghaddam A Mohammadian S Vazini H Taghadosi M Esmaeili S-A Mardani F. Macrophage Plasticity, Polarization, and Function in Health and Disease. J Cell Physiol (2018) 233:6425–40. 10.1002/jcp.26429 PMC13482560 29319160

[83] Ostuni R Kratochvill F Murray PJ Natoli G. Macrophages and Cancer: From Mechanisms to Therapeutic Implications. Trends Immunol (2015) 36:229–39. 10.1016/j.it.2015.02.004 25770924

[84] Geissmann F Manz MG Jung S Sieweke MH Merad M Ley K. Development of Monocytes, Macrophages, and Dendritic Cells. Science (2010) 327:656–61. 10.1126/science.1178331 PMC2887389 20133564

[85] Epelman S Lavine KJ Randolph GJ. Origin and Functions of Tissue Macrophages. Immunity (2014) 41:21–35. 10.1016/j.immuni.2014.06.013 25035951 PMC4470379

[86] Wu MH Lee WJ Hua KT Kuo ML Lin MT. Macrophage Infiltration Induces Gastric Cancer Invasiveness by Activating the β-Catenin Pathway. PLoS One (2015) 10(7):e0134122. 10.1371/journal.pone.0134122 26226629 PMC4520459

[87] Tiainen S Tumelius R Rilla K Hämäläinen K Tammi M Tammi R. High Numbers of Macrophages, Especially M2-Like (CD163-Positive), Correlate With Hyaluronan Accumulation and Poor Outcome in Breast Cancer. Histopathology (2015) 66(6):873–83. 10.1111/his.12607 25387851

[88] Wang H Hu Wm Xia Zj Liang Y Lu Y Lin Sx. High numbers of CD163+ tumor-associated macrophages correlate with poor prognosis in multiple myeloma patients receiving bortezomib-based regimens. J Cancer (2019) 10(14):3239–45. 10.7150/jca.30102 PMC6603386 31289595

[89] Macciò A Gramignano G Cherchi MC Tanca L Melis L Madeddu C. Role of M1-Polarized Tumor-Associated Macrophages in the Prognosis of Advanced Ovarian Cancer Patients. Sci Rep (2020) 10(1):6096. 10.1038/s41598-020-63276-1 32269279 PMC7142107

[90] Hegab AE Ozaki M Kagawa S Hamamoto J Yasuda H Naoki K. Tumor Associated Macrophages Support the Growth of FGF9-induced Lung Adenocarcinoma by Multiple Mechanisms. Lung Cancer (2018) 119:25–35. 10.1016/j.lungcan.2018.02.015 29656749

[91] Sahraei M Chaube B Liu Y Sun J Kaplan A Price NL. Suppressing miR-21 Activity in Tumor-Associated Macrophages Promotes an Antitumor Immune Response. J Clin Invest (2019) 129:5518–36. 10.1172/JCI127125 PMC6877327 31710308

[92] Chittezhath M Dhillon MK Lim JY Laoui D Shalova IN Teo YL. Molecular Profiling Reveals a Tumor-Promoting Phenotype of Monocytes and Macrophages in Human Cancer Progression. Immunity (2014) 41:815—29. 10.1016/j.immuni.2014.09.014 25453823

[93] Asgharzadeh S Salo JA Ji L Oberthuer A Fischer M Berthold F. Clinical Significance of Tumor-Associated Inflammatory Cells in Metastatic Neuroblastoma. J Clin Oncol (2012) 30:3525—32. 10.1200/jco.2011.40.9169 22927533 PMC3675667

[94] Shabo I Stål O Olsson H Doré S Svanvik J. Breast Cancer Expression of CD163, a Macrophage Scavenger Receptor, is Related to Early Distant Recurrence and Reduced Patient Survival. Int J Cancer (2008) 123:780–6. 10.1002/ijc.23527 18506688

[95] Cho H Seo Y Loke KM Kim S-W Oh S-M Kim J-H. Cancer-Stimulated CAFs Enhance Monocyte Differentiation and Protumoral Tam Activation *Via* IL6 and GM-CSF Secretion. Clin Cancer Res (2018) 24:5407–21. 10.1158/1078-0432.CCR-18-0125 29959142

[96] Marcuzzi E Angioni R Molon B Calì B. Correction: Marcuzzi, E., Et Al. Chemokines and Chemokine Receptors: Orchestrating Tumor Metastasization. Int J Mol Sci (2019) 20:96. 10.3390/ijms20112651 PMC6600455 31146450

[97] Gustavsson M Zheng Y Handel TM. Production of Chemokine/Chemokine Receptor Complexes for Structural Biophysical Studies. Methods Enzymol (2016) 570:233–60. 10.1016/bs.mie.2015.10.003 PMC4770566 26921949

[98] Henze A-T Mazzone M. The Impact of Hypoxia on Tumor-Associated Macrophages. J Clin Invest (2016) 126:3672–9. 10.1172/JCI84427 PMC5096805 27482883

[99] Nasrollahzadeh E Razi S Keshavarz-Fathi M Mazzone M Rezaei N. Pro-Tumorigenic Functions of Macrophages At the Primary, Invasive and Metastatic Tumor Site. Cancer Immunol Immunother (2020) 69:1673–97. 10.1007/s00262-020-02616-6 PMC11027658 32500231

[100] Yeo E-J Cassetta L Qian B-Z Lewkowich I Li J Stefater 3JA. Myeloid WNT7b Mediates the Angiogenic Switch and Metastasis in Breast Cancer. Cancer Res (2014) 74:2962–73. 10.1158/0008-5472.CAN-13-2421 PMC4137408 24638982

[101] Gordon SR Maute RL Dulken BW Hutter G George BM McCracken MN. PD-1 Expression by Tumour-Associated Macrophages Inhibits Phagocytosis and Tumour Immunity. Nature (2017) 545:495–9. 10.1038/nature22396 PMC5931375 28514441

[102] Katsuya Y Horinouchi H Asao T Kitahara S Goto Y Kanda S. Expression of Programmed Death 1 (PD-1) and its Ligand (PD-L1) in Thymic Epithelial Tumors: Impact on Treatment Efficacy and Alteration in Expression After Chemotherapy. Lung Cancer (2016) 99:4–10. 10.1016/j.lungcan.2016.05.007 27565906

[103] Jarnicki AG Lysaght J Todryk S Mills KHG. Suppression of Antitumor Immunity by IL-10 and TGF-β-Producing T Cells Infiltrating the Growing Tumor: Influence of Tumor Environment on the Induction of CD4^+^ and CD8^+^ Regulatory T Cells. J Immunol (2006) 177:896–904. 10.4049/jimmunol.177.2.896 16818744

[104] Jetten N Verbruggen S Gijbels MJ Post MJ De Winther MPJ Donners MMPC. Anti-Inflammatory M2, But Not Pro-Inflammatory M1 Macrophages Promote Angiogenesis. Vivo Angiogenesis (2014) 17:109–18. 10.1007/s10456-013-9381-6 24013945

[105] Di Caro G Cortese N Castino GF Grizzi F Gavazzi F Ridolfi C. Dual Prognostic Significance of Tumour-Associated Macrophages in Human Pancreatic Adenocarcinoma Treated or Untreated With Chemotherapy. Gut (2016) 65:1710–20. 10.1136/gutjnl-2015-309193 26156960

[106] Guo Z Song J Hao J Zhao H Du X Li E. M2 Macrophages Promote NSCLC Metastasis by Upregulating CRYAB. Cell Death Dis (2019) 10:377. 10.1038/s41419-019-1618-x 31097690 PMC6522541

[107] Zhang H Wang X Shen Z Xu J Qin J Sun Y. Infiltration of Diametrically Polarized Macrophages Predicts Overall Survival of Patients With Gastric Cancer After Surgical Resection. Gastric Cancer (2015) 18:740–50. 10.1007/s10120-014-0422-7 25231913

[108] Ren C-X Leng R-X Fan Y-G Pan H-F Li B-Z Wu C-H. Intratumoral and Peritumoral Expression of CD68 and CD206 in Hepatocellular Carcinoma and Their Prognostic Value. Oncol Rep (2017) 38:886–98. 10.3892/or.2017.5738 PMC5561967 28656201

[109] Zheng X Weigert A Reu S Guenther S Mansouri S Bassaly B. Spatial Density and Distribution of Tumor-Associated Macrophages Predict Survival in Non-Small Cell Lung Carcinoma. Cancer Res (2020) 80:4414—4425. 10.1158/0008-5472.can-20-0069 32699134

[110] Pelekanou V Villarroel-Espindola F Schalper KA Pusztai L Rimm DL. Cd68, CD163, and Matrix Metalloproteinase 9 (MMP-9) Co-Localization in Breast Tumor Microenvironment Predicts Survival Differently in ER-positive and -Negative Cancers. Breast Cancer Res (2018) 20:154. 10.1186/s13058-018-1076-x 30558648 PMC6298021

[111] Qian B-Z Pollard JW. Macrophage Diversity Enhances Tumor Progression and Metastasis. Cell (2010) 141:39–51. 10.1016/j.cell.2010.03.014 20371344 PMC4994190

[112] Patsialou A Wyckoff J Wang Y Goswami S Stanley ER Condeelis JS. Invasion of Human Breast Cancer Cells *In Vivo* Requires Both Paracrine and Autocrine Loops Involving the Colony-Stimulating Factor-1 Receptor. Cancer Res (2009) 69:9498–506. 10.1158/0008-5472.CAN-09-1868 PMC2794986 19934330

[113] Zheng P Luo Q Wang W Li J Wang T Wang P. Tumor-Associated Macrophages-Derived Exosomes Promote the Migration of Gastric Cancer Cells by Transfer of Functional Apolipoprotein E. Cell Death Dis (2018) 9:434. 10.1038/s41419-018-0465-5 29567987 PMC5864742

[114] Binenbaum Y Fridman E Yaari Z Milman N Schroeder A Ben David G. Transfer of miRNA in Macrophage-Derived Exosomes Induces Drug Resistance in Pancreatic Adenocarcinoma. Cancer Res (2018) 78:5287–99. 10.1158/0008-5472.CAN-18-0124 30042153

[115] Lee K Park JW Lee K Cho S Kwon Y-H Kim MJ. Adjuvant Chemotherapy Does Not Provide Survival Benefits to Elderly Patients With Stage II Colon Cancer. Sci Rep (2019) 9:11846. 10.1038/s41598-019-48197-y 31413354 PMC6694195

[116] André T de Gramont A Vernerey D Chibaudel B Bonnetain F Tijeras-Raballand A. Adjuvant Fluorouracil, Leucovorin, and Oxaliplatin in Stage II to III Colon Cancer: Updated 10-Year Survival and Outcomes According to BRAF Mutation and Mismatch Repair Status of the MOSAIC Study. J Clin Oncol (2015) 33:4176–87. 10.1200/JCO.2015.63.4238 26527776

[117] Feng Q Chang W Mao Y He G Zheng P Tang W. Tumor-Associated Macrophages as Prognostic and Predictive Biomarkers for Postoperative Adjuvant Chemotherapy in Patients With Stage Ii Colon Cancer. Clin Cancer Res (2019) 25:3896–907. 10.1158/1078-0432.CCR-18-2076 30988081

[118] Argyle D Kitamura T. Targeting Macrophage-Recruiting Chemokines as a Novel Therapeutic Strategy to Prevent the Progression of Solid Tumors. Front Immunol (2018) 9:2629. 10.3389/fimmu.2018.02629 30483271 PMC6243037

[119] Loberg RD Ying C Craig M Day LL Sargent E Neeley C. Targeting CCL2 With Systemic Delivery of Neutralizing Antibodies Induces Prostate Cancer Tumor Regression In Vivo. Cancer Res (2007) 67:9417–24. 10.1158/0008-5472.CAN-07-1286 17909051

[120] Tu MM Abdel-Hafiz HA Jones RT Jean A Hoff KJ Duex JE. Inhibition of the CCL2 Receptor, CCR2, Enhances Tumor Response to Immune Checkpoint Therapy. Commun Biol (2020) 3:720. 10.1038/s42003-020-01441-y 33247183 PMC7699641

[121] Nywening TM Wang-Gillam A Sanford DE Belt BA Panni RZ Cusworth BM. Targeting Tumour-Associated Macrophages With CCR2 Inhibition in Combination With FOLFIRINOX in Patients With Borderline Resectable and Locally Advanced Pancreatic Cancer: A Single-Centre, Open-Label, Dose-Finding, non-Randomised, Phase 1b Trial. Lancet Oncol (2016) 17:651–62. 10.1016/S1470-2045(16)00078-4 PMC5407285 27055731

[122] Frankenberger C Rabe D Bainer R Sankarasharma D Chada K Krausz T. Metastasis Suppressors Regulate the Tumor Microenvironment by Blocking Recruitment of Prometastatic Tumor-Associated Macrophages. Cancer Res (2015) 75:4063–73. 10.1158/0008-5472.CAN-14-3394 PMC4592465 26238785

[123] DeNardo DG Brennan DJ Rexhepaj E Ruffell B Shiao SL Madden SF. Leukocyte Complexity Predicts Breast Cancer Survival and Functionally Regulates Response to Chemotherapy. Cancer Discovery (2011) 1:54–67. 10.1158/2159-8274.CD-10-0028 22039576 PMC3203524

[124] Strachan DC Ruffell B Oei Y Bissell MJ Coussens LM Pryer N. CSF1R Inhibition Delays Cervical and Mammary Tumor Growth in Murine Models by Attenuating the Turnover of Tumor-Associated Macrophages and Enhancing Infiltration by CD8(+) T Cells. Oncoimmunology (2013) 2:e26968–8. 10.4161/onci.26968 PMC3902121 24498562

[125] Xu J Escamilla J Mok S David J Priceman S West B. CSF1R Signaling Blockade Stanches Tumor-Infiltrating Myeloid Cells and Improves the Efficacy of Radiotherapy in Prostate Cancer. Cancer Res (2013) 73:2782–94. 10.1158/0008-5472.CAN-12-3981 PMC4097014 23418320

[126] Anfray C Ummarino A Andón FT Allavena P. Current Strategies to Target Tumor-Associated-Macrophages to Improve Anti-Tumor Immune Responses. Cells (2020) 9:1–24. 10.3390/cells9010046 PMC7017001 31878087

[127] Zhang R Qi F Zhao F Li G Shao S Zhang X. Cancer-Associated Fibroblasts Enhance Tumor-Associated Macrophages Enrichment and Suppress NK Cells Function in Colorectal Cancer. Cell Death Dis (2019) 10:273. 10.1038/s41419-019-1435-2 30894509 PMC6426970

[128] Comito G Giannoni E Segura CP Barcellos-de-Souza P Raspollini MR Baroni G. Cancer-Associated Fibroblasts and M2-polarized Macrophages Synergize During Prostate Carcinoma Progression. Oncogene (2014) 33:2423–31. 10.1038/onc.2013.191 23728338

[129] Fujii N Shomori K Shiomi T Nakabayashi M Takeda C Ryoke K. Cancer-Associated Fibroblasts and CD163-positive Macrophages in Oral Squamous Cell Carcinoma: Their Clinicopathological and Prognostic Significance. J Oral Pathol Med (2012) 41:444–51. 10.1111/j.1600-0714.2012.01127.x 22296275

[130] Larsson K Kock A Idborg H Arsenian Henriksson M Martinsson T Johnsen JI. Cox/mPGES-1/PGE2 Pathway Depicts an Inflammatory-Dependent High-Risk Neuroblastoma Subset. Proc Natl Acad Sci USA (2015) 112:8070–5. 10.1073/pnas.1424355112 PMC4491767 26080408

[131] An Y Liu F Chen Y Yang Q. Crosstalk Between Cancer-Associated Fibroblasts and Immune Cells in Cancer. J Cell Mol Med (2020) 24:13–24. 10.1111/jcmm.14745 31642585 PMC6933413

[132] Tokuda K Morine Y Miyazaki K Yamada S Saito Y Nishi M. The Interaction Between Cancer Associated Fibroblasts and Tumor Associated Macrophages Via the Osteopontin Pathway in the Tumor Microenvironment of Hepatocellular Carcinoma. Oncotarget (2021) 12(4):333–43. 10.18632/oncotarget.27881 PMC7899554 33659044

[133] Adams DL Martin SS Alpaugh RK Charpentier M Tsai S Bergan RC. Circulating Giant Macrophages as a Potential Biomarker of Solid Tumors. Proc Natl Acad Sci USA (2014) 111:3514–9. 10.1073/pnas.1320198111 PMC3948254 24550495

[134] Condeelis J Pollard JW. Macrophages: Obligate Partners for Tumor Cell Migration, Invasion, and Metastasis. Cell (2006) 124:263–6. 10.1016/j.cell.2006.01.007 16439202

[135] Brown JM Recht L Strober S. The Promise of Targeting Macrophages in Cancer Therapy. Clin Cancer Res (2017) 23:3241–50. 10.1158/1078-0432.CCR-16-3122 PMC5529121 28341752

[136] Lee H-O Mullins SR Franco-Barraza J Valianou M Cukierman E Cheng JD. FAP-Overexpressing Fibroblasts Produce an Extracellular Matrix That Enhances Invasive Velocity and Directionality of Pancreatic Cancer Cells. BMC Cancer (2011) 11:245. 10.1186/1471-2407-11-245 21668992 PMC3141768

[137] Paolillo M Schinelli S. Extracellular Matrix Alterations in Metastatic Processes. Int J Mol Sci (2019) 20:4947. 10.3390/ijms20194947 PMC6802000 31591367

[138] Vindin H Mithieux SM Weiss AS. Elastin Architecture. Matrix Biol (2019) 84:4—16. 10.1016/j.matbio.2019.07.005 31301399

[139] Oxford JT Reeck JC Hardy MJ. Extracellular Matrix in Development and Disease. Int J Mol Sci (2019) 20:205. 10.3390/ijms20010205 PMC6337388 30626024

[140] Burmakin M van Wieringen T Olsson PO Stuhr L Åhgren A Heldin C-H. Imatinib Increases Oxygen Delivery in Extracellular Matrix-Rich But Not in Matrix-Poor Experimental Carcinoma. J Transl Med (2017) 15:47. 10.1186/s12967-017-1142-7 28231806 PMC5324310

[141] Olsson PO Gustafsson R In 't Zandt R Friman T Maccarana M Tykesson E. The Tyrosine Kinase Inhibitor Imatinib Augments Extracellular Fluid Exchange and Reduces Average Collagen Fibril Diameter in Experimental Carcinoma. Mol Cancer Ther (2455) 2016) 15:2455–64. 10.1158/1535-7163.MCT-16-0026 27474147

[142] Vidak E Javoršek U Vizovišek M Turk B. Cysteine Cathepsins and Their Extracellular Roles: Shaping the Microenvironment. Cells (2019) 8:264. 10.3390/cells8030264 PMC6468544 30897858

[143] Afik R Zigmond E Vugman M Klepfish M Shimshoni E Chor MP. Tumor Macrophages are Pivotal Constructors of Tumor Collagenous Matrix. J Exp Med (2016) 213:2315–31. 10.1084/jem.20151193 PMC5068227 27697834

[144] Mongiat M Andreuzzi E Tarticchio G Paulitti A. Extracellular Matrix, a Hard Player in Angiogenesis. Int J Mol Sci (2016) 17:1822. 10.3390/ijms17111822 PMC5133823 27809279

[145] Bonnans C Chou J Werb Z. Remodelling the Extracellular Matrix in Development and Disease. Nat Rev Mol Cell Biol (2014) 15:786–801. 10.1038/nrm3904 25415508 PMC4316204

[146] Eiro N Carrión JF Cid S Andicoechea A García-Muñiz JL González LO. Toll-Like Receptor 4 and Matrix Metalloproteases 11 and 13 as Predictors of Tumor Recurrence and Survival in Stage Ii Colorectal Cancer. Pathol Oncol Res (2019) 25:1589—97. 10.1007/s12253-019-00611-6 30710321

[147] Cancemi P Buttacavoli M Roz E Feo S. Expression of Alpha-Enolase (Eno1), Myc Promoter-Binding Protein-1 (Mbp-1) and Matrix Metalloproteinases (MMP-2 and MMP-9) Reflect the Nature and Aggressiveness of Breast Tumors. Int J Mol Sci (2019) 20:3952. 10.3390/ijms20163952 PMC6720302 31416219

[148] Honda H Takamura M Yamagiwa S Genda T Horigome R Kimura N. Overexpression of a Disintegrin and Metalloproteinase 21 is Associated With Motility, Metastasis, and Poor Prognosis in Hepatocellular Carcinoma. Sci Rep (2017) 7:15485. 10.1038/s41598-017-15800-z 29138461 PMC5686078

[149] Netea-Maier RT Smit JWA Netea MG. Metabolic Changes in Tumor Cells and Tumor-Associated Macrophages: A Mutual Relationship. Cancer Lett (2018) 413:102–9. 10.1016/j.canlet.2017.10.037 29111350

[150] Lakins MA Ghorani E Munir H Martins CP Shields JD. Cancer-Associated Fibroblasts Induce Antigen-Specific Deletion of CD8+ T Cells to Protect Tumour Cells. Nat Commun (2018) 9:948. 10.1038/s41467-018-03347-0 29507342 PMC5838096

[151] Iessi E Logozzi M Mizzoni D Di Raimo R Supuran CT Fais S. Rethinking the Combination of Proton Exchanger Inhibitors in Cancer Therapy. Metab (2018) 8:1–20. 10.3390/metabo8010002 PMC5875992 29295495

[152] Singh S Lomelino CL Mboge MY Frost SC McKenna R. Cancer Drug Development of Carbonic Anhydrase Inhibitors Beyond the Active Site. Molecules (2018) 23:1–22. 10.3390/molecules23051045 PMC6099549 29710858

[153] Winkler J Abisoye-Ogunniyan A Metcalf KJ Werb Z. Concepts of Extracellular Matrix Remodelling in Tumour Progression and Metastasis. Nat Commun (2020) 11:5120. 10.1038/s41467-020-18794-x 33037194 PMC7547708

[154] Havel JJ Chowell D Chan TA. The Evolving Landscape of Biomarkers for Checkpoint Inhibitor Immunotherapy. Nat Rev Cancer (2019) 19:133–50. 10.1038/s41568-019-0116-x PMC6705396 30755690

[155] Tran L Theodorescu D. Determinants of Resistance to Checkpoint Inhibitors. Int J Mol Sci (2020) 21:1594. 10.3390/ijms21051594 PMC7084564 32111080

[156] Gorchs L Fernández Moro C Bankhead P Kern KP Sadeak I Meng Q. Human Pancreatic Carcinoma-Associated Fibroblasts Promote Expression of Co-inhibitory Markers on CD4(+) and CD8(+) T-Cells. Front Immunol (2019) 10:847. 10.3389/fimmu.2019.00847 31068935 PMC6491453

[157] Chen S Giannakou A Wyman S Gruzas J Golas J Zhong W. Cancer-Associated Fibroblasts Suppress SOX2-induced Dysplasia in a Lung Squamous Cancer Coculture. Proc Natl Acad Sci (2018) 115:E11671–80. 10.1073/pnas.1803718115 PMC6294935 30487219

[158] Zhou W Guo S Liu M Burow ME Wang G. Targeting CXCL12/CXCR4 Axis in Tumor Immunotherapy. Curr Med Chem (2019) 26:3026–41. 10.2174/0929867324666170830111531 PMC5949083 28875842

[159] Martinez-Outschoorn UE Prisco M Ertel A Tsirigos A Lin Z Pavlides S. Ketones and Lactate Increase Cancer Cell “Stemness,” Driving Recurrence, Metastasis and Poor Clinical Outcome in Breast Cancer: Achieving Personalized Medicine. via Metabolo-Genom Cell Cycle (2011) 10:1271–86. 10.4161/cc.10.8.15330 PMC3117136 21512313

[160] Komohara Y Takeya M. Cafs and TAMs: Maestros of the Tumour Microenvironment. J Pathol (2017) 241:313–5. 10.1002/path.4824 27753093

[161] Kumar V Donthireddy L Marvel D Condamine T Wang F Lavilla-Alonso S. Cancer-Associated Fibroblasts Neutralize the Anti-tumor Effect of CSF1 Receptor Blockade by Inducing Pmn-Mdsc Infiltration of Tumors. Cancer Cell (2017) 32:654+. 10.1016/j.ccell.2017.10.005 29136508 PMC5827952

[162] Mariathasan S Turley SJ Nickles D Castiglioni A Yuen K Wang Y. Tgfβ Attenuates Tumour Response to PD-L1 Blockade by Contributing to Exclusion of T Cells. Nature (2018) 554:544–8. 10.1038/nature25501 PMC6028240 29443960

[163] Liu B Guo H Xu J Qin T Guo Q Gu N. Elimination of Tumor by CD47/PD-L1 Dual-Targeting Fusion Protein That Engages Innate and Adaptive Immune Responses. MAbs (2018) 10:315–24. 10.1080/19420862.2017.1409319 29182441 PMC5825205

[164] Sockolosky JT Dougan M Ingram JR Ho CCM Kauke MJ Almo SC. Durable Antitumor Responses to CD47 Blockade Require Adaptive Immune Stimulation. Proc Natl Acad Sci USA (2016) 113:E2646—54. 10.1073/pnas.1604268113 27091975 PMC4868409

[165] Shi R Chai Y Duan X Bi X Huang Q Wang Q. The Identification of a CD47-blocking “Hotspot” and Design of a CD47/PD-L1 Dual-Specific Antibody With Limited Hemagglutination. Signal Transduct Target Ther (2020) 5:16. 10.1038/s41392-020-0121-2 32296041 PMC7058617

[166] Schmid BC Oehler MK. Improvements in Progression-Free and Overall Survival Due to the Use of Anti-Angiogenic Agents in Gynecologic Cancers. Curr Treat Options Oncol (2015) 16:318. 10.1007/s11864-014-0318-0 25750175

[167] Hofheinz R Ronellenfitsch U Kubicka S Falcone A Burkholder I Hacker U. Treatment With Antiangiogenic Drugs in Multiple Lines in Patients With Metastatic Colorectal Cancer: Meta-Analysis of Randomized Trials. Gastroenterol Res Pract (2016) 2016:9189483. 10.1155/2016/9189483 27656206 PMC5021498

[168] Mawalla B Yuan X Luo X Chalya PL. Treatment Outcome of Anti-Angiogenesis Through VEGF-pathway in the Management of Gastric Cancer: A Systematic Review of Phase II and III Clinical Trials. BMC Res Notes (2018) 11:21. 10.1186/s13104-018-3137-8 29329598 PMC5767044

[169] Loges S Schmidt T Carmeliet P. Mechanisms of Resistance to Anti-Angiogenic Therapy and Development of Third-Generation Anti-Angiogenic Drug Candidates. Genes Cancer (2010) 1:12–25. 10.1177/1947601909356574 21779425 PMC3092176

[170] Teleanu RI Chircov C Grumezescu AM Teleanu DM. Tumor Angiogenesis and Anti-Angiogenic Strategies for Cancer Treatment. J Clin Med (2019) 9:84. 10.3390/jcm9010084 PMC7020037 31905724

[171] Chiang C-F Chao T-T Su Y-F Hsu C-C Chien C-Y Chiu K-C. Metformin-Treated Cancer Cells Modulate Macrophage Polarization Through AMPK-NF-κb Signaling. Oncotarget (2017) 8:20706–18. 10.18632/oncotarget.14982 PMC5400538 28157701

[172] Di Matteo S Nevi L Overi D Landolina N Faccioli J Giulitti F. Metformin Exerts Anti-Cancerogenic Effects and Reverses Epithelial-to-Mesenchymal Transition Trait in Primary Human Intrahepatic Cholangiocarcinoma Cells. Sci Rep (2021) 11:2557. 10.1038/s41598-021-81172-0 33510179 PMC7844056

[173] Kamarudin MNA Sarker MMR Zhou J-R Parhar I. Metformin in Colorectal Cancer: Molecular Mechanism, Preclinical and Clinical Aspects. J Exp Clin Cancer Res (2019) 38:491. 10.1186/s13046-019-1495-2 31831021 PMC6909457

[174] Winer A Adams S Mignatti P. Matrix Metalloproteinase Inhibitors in Cancer Therapy: Turning Past Failures Into Future Successes. Mol Cancer Ther (2018) 17:1147–55. 10.1158/1535-7163.mct-17-0646 29735645 PMC5984693

[175] Laklai H Miroshnikova YA Pickup MW Collisson EA Kim GE Barrett AS. Genotype Tunes Pancreatic Ductal Adenocarcinoma Tissue Tension to Induce Matricellular Fibrosis and Tumor Progression. Nat Med (2016) 22:497–505. 10.1038/nm.4082 27089513 PMC4860133

[176] Heichler C Scheibe K Schmied A Geppert CI Schmid B Wirtz S. STAT3 Activation Through IL-6/IL-11 in Cancer-Associated Fibroblasts Promotes Colorectal Tumour Development and Correlates With Poor Prognosis. Gut (2020) 69:1269–82. 10.1136/gutjnl-2019-319200 31685519

[177] Zou S Tong Q Liu B Huang W Tian Y Fu X. Targeting STAT3 in Cancer Immunotherapy. Mol Cancer (2020) 19:145. 10.1186/s12943-020-01258-7 32972405 PMC7513516

